# Supplementary material for: Lipid nanoparticle-encapsulated DOCK11-siRNA efficiently reduces hepatitis B virus cccDNA level in infected mice
Source: Mol Ther Methods Clin Dev. 2024 Jun 24;32(3):101289. doi: 10.1016/j.omtm.2024.101289 (PMC11300937; doi:10.1016/j.omtm.2024.101289)
Supplement: Document S1. Figures S1–S11 [file mmc1.pdf]

OMTM, Volume *32*

Supplemental information

Lipid nanoparticle-encapsulated DOCK11-siRNA efficiently reduces hepatitis B virus cccDNA level in infected mice

Hikari Okada, Takeharu Sakamoto, Kouki Nio, Yingyi Li, Kazuyuki Kuroki, Saiho Sugimoto, Tetsuro Shimakami, Nobuhide Doi, Masao Honda, Motoharu Seiki, Shuichi Kaneko, and Taro Yamashita

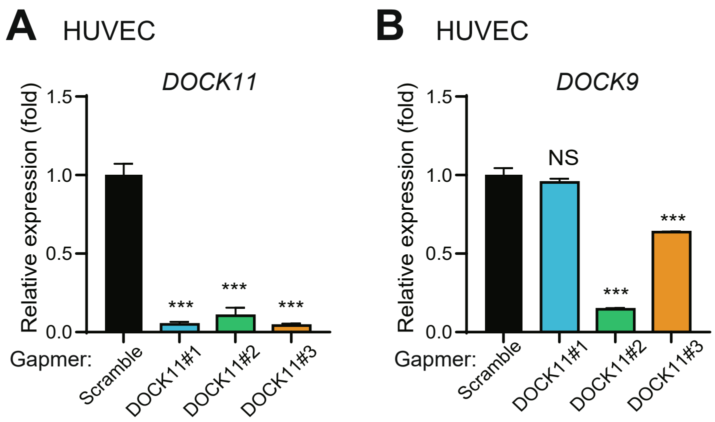

Figure S1. Comparative expression analyses of DOCK11 and DOCK9 in HUVEC after gapmer transfection.
(A, B) Expression levels of DOCK11 (A) and DOCK9 mRNA (B) in HUVEC transfected with gapmer scramble and DOCK11#1-3. Data are presented as the mean (SD) (n = 3) and analyzed using the two-sided unpaired t-test with Welch' s correction. ***P < 0.001.

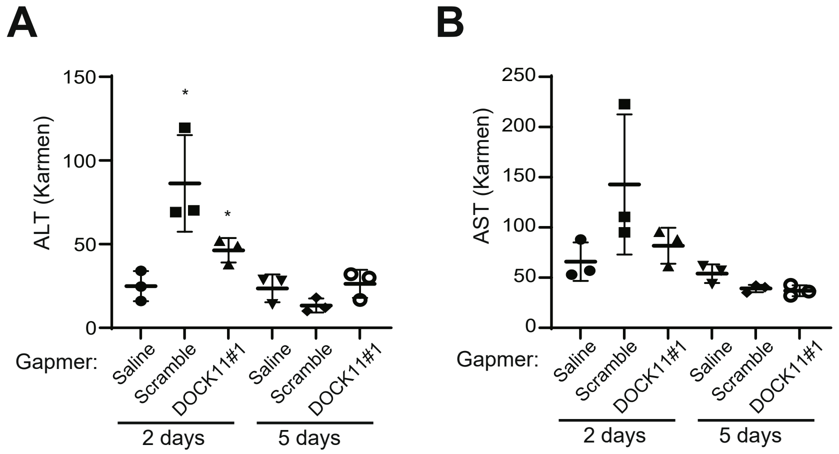

Figure S2. Hepatotoxic effects of gapmers in mice.
(A, B) Serum AST (A) and ALT (B) levels in wild-type mice 2 and 5 days after gapmer injection. Data are presented as the mean (SD) (n = 3) and analyzed using the Mann–Whitney U-test. *P < 0.05.

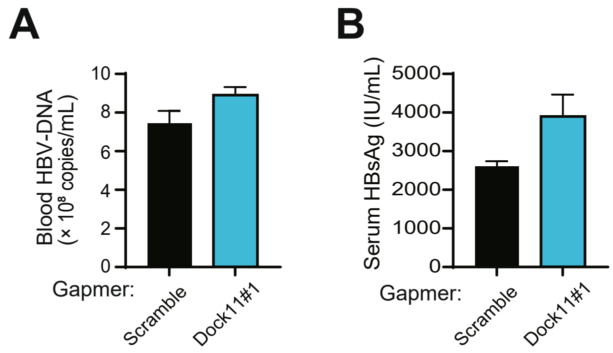

Figure S3. Administration of gapmer DOCK11#1 does not reduce blood HBV-DNA and serum HBsAg levels in HBV-infected human liver chimeric mice. (A, B) Comparative levels of blood HBV-DNA (A) and serum HBsAg (B) in HBV-chronic infected human liver chimeric mice after repeated administration of gapmer scramble and DOCK11#1. Data are presented as the mean (SD) (n = 3).

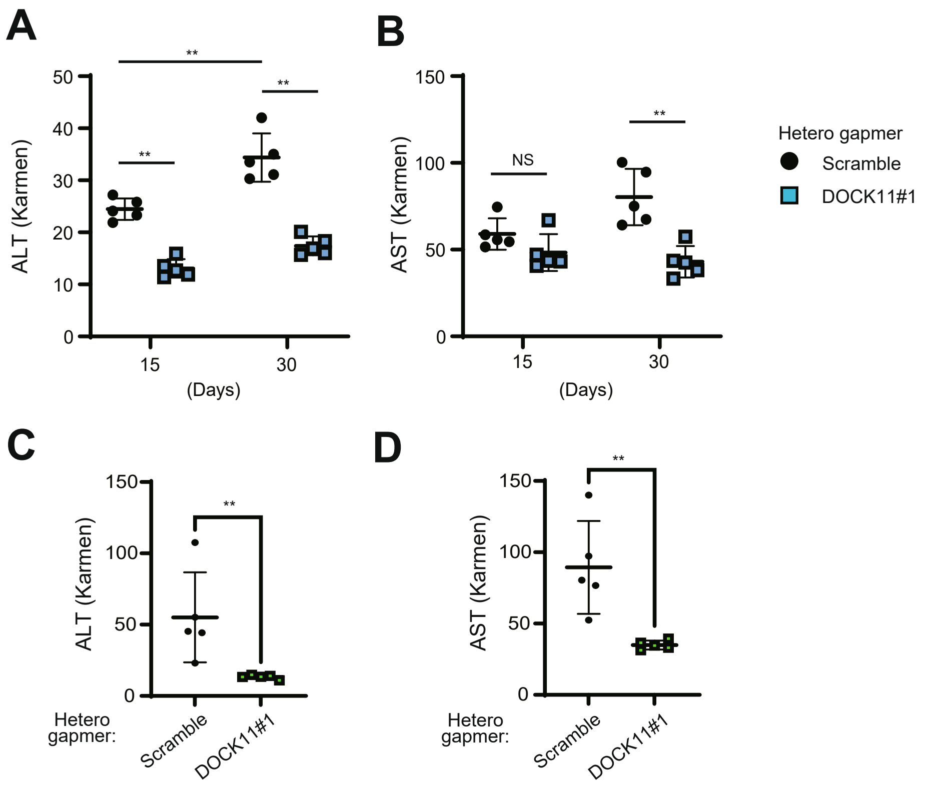

Figure S4. Hepatotoxic effects of hetero-gapmers in wild-type and AAV8-HBV1.3mer-infected mice.
(A, B) Serum ALT (A) and AST (B) levels on day 5 after administration of hetero-gapmer to AAV8-HBV1.3mer infected early and chronic phase model mice.
(C, D) Serum ALT (C) and AST (D) levels in wild-type mice 5 days after hetero-gapmer administration.
Data are presented as the mean (SD) (n = 5) and analyzed using the Mann-Whitney U-test. *P < 0.05, *P < 0.01.

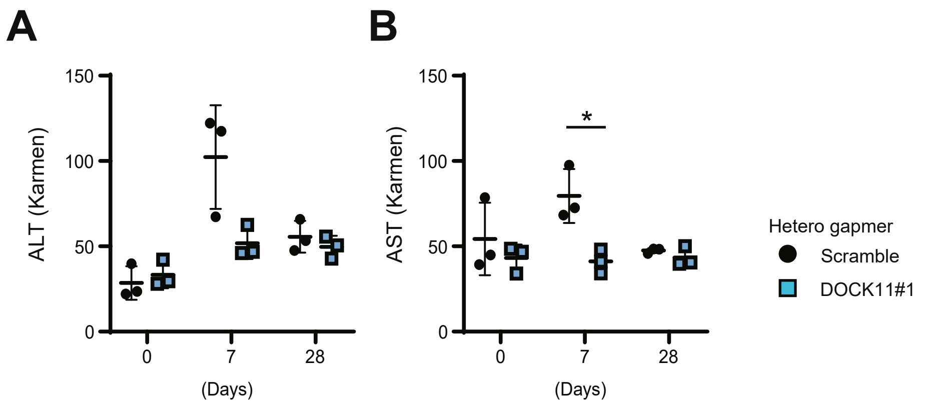

Figure S5. Serum ALT and AST levels to evaluate the hepatotoxicity of hetero-gapmer DOCK11#1 in HBV-infected human liver chimeric mice. (A, B) Comparison of serum ALT (A) and serum AST (B) levels on days 0, 7, and 28 in HBV chronically infected human liver chimeric mice after repeated administration of hetero-gapmer scamble and DOCK11#1. Data are presented as the mean (SD) (n = 3) and analyzed using the Mann-Whitney U-test. *P < 0.05.

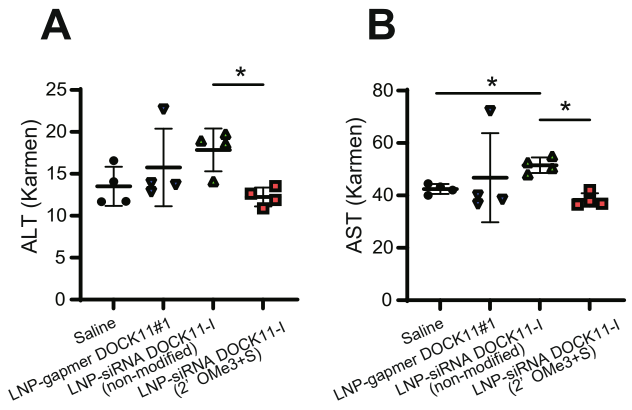

Figure S6. Verification of tropism of LNP-nucleic acid modality in AAV8-HBV1.3mer-infected model mice.
(A, B) Serum ALT (A) and AST (B) levels 7 days after administration of LNP-gapmer, LNP-siRNA non-modified, and LNP-siRNA 2' OMe+S targeting the DOCK11 mRNA in AAV8-HBV1.3mer-infected model mice.
Data are presented as the mean (SD) (n = 4) and analyzed using the Mann-Whitney U-test. *$P < 0.05$.

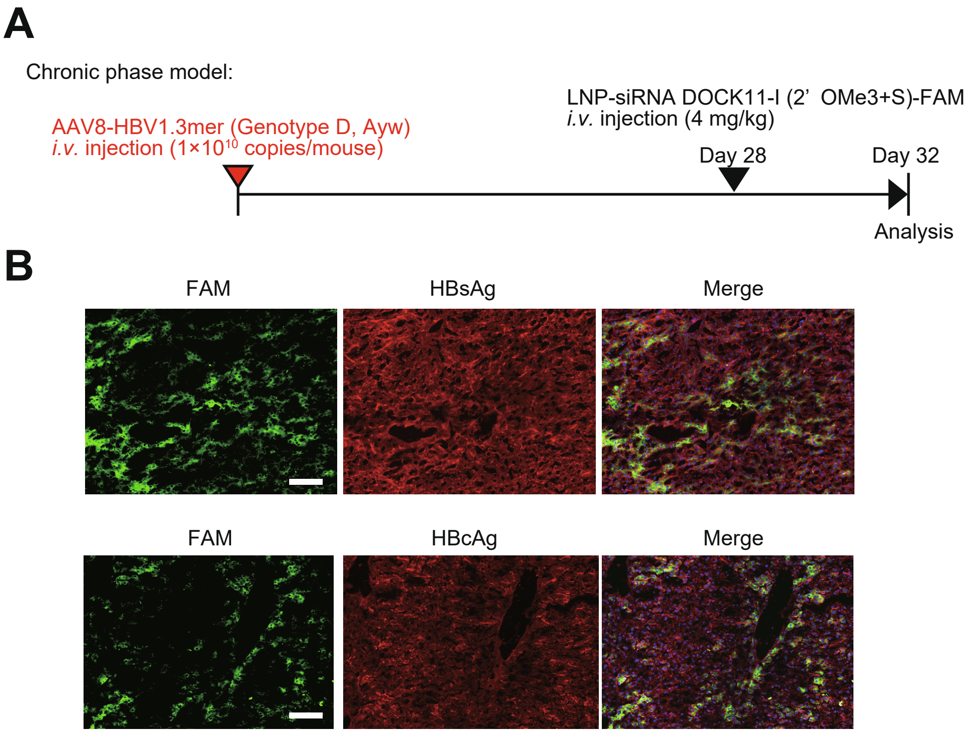

Figure S7. Verification of tropism of LNP-siRNA DOCK11-l in AAV8-HBV1.3mer infected model mice.
(A, B) Schedule for verifying whether LNP-siRNA DOCK11-l (2' OMe3+S) is taken up into HBV-positive hepatocytes of AAV8-HBV1.3mer infection model mice (A). Double fluorescent staining of the liver tissue with Alexa594-labeled HBsAg or HBcAg 5 days after administration of FAM-labeled LNP-siRNA DOCK11-l (B).

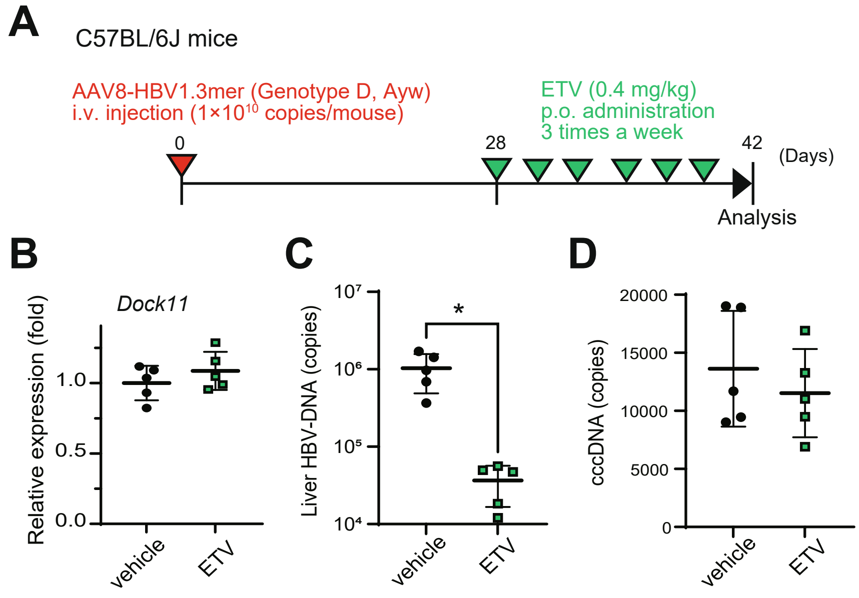

Figure S8. Entecavir (ETV) reduces liver HBV-DNA but not cccDNA levels in AV8-HBV1.3mer infected mice.
(A) The schedule for ETV administration in mouse models of AAV8-HBV1.3mer chronic infection. (B, C, D) The relative expression levels of DOCK11 (B), and HBV DNA (C) and cccDNA (D) levels in the liver of AAV8-HBV1.3mer-infected mice treated with entecavir. Data are presented as the mean (SD) (n = 5) and analyzed using the Mann–Whitney U-test. *$P < 0.05$.

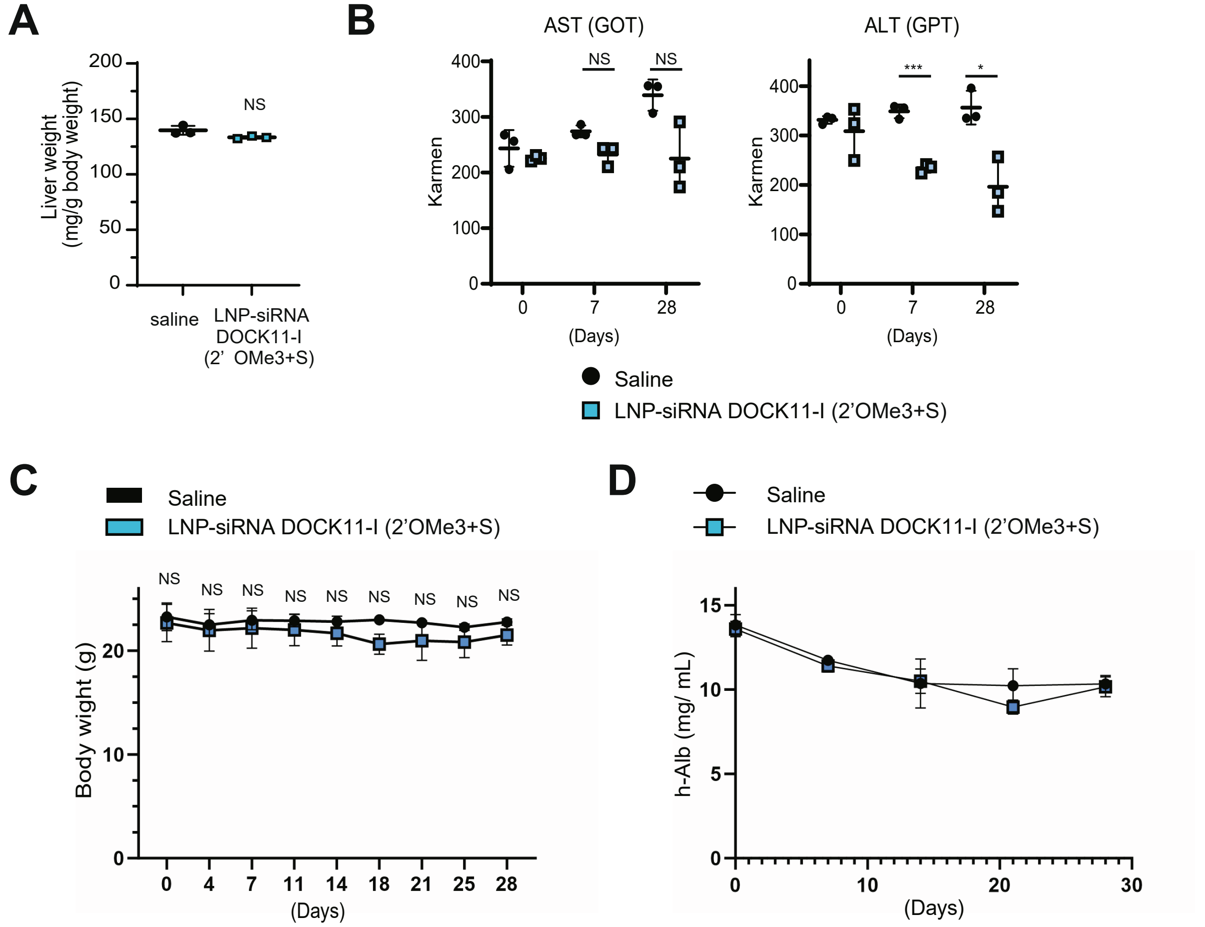

Figure S9. LNP-siRNA DOCK11-I (2' OMe3+S) exhibits no toxicity in HBV-infected human liver chimeric mice.
The effects of LNP-siRNA DOCK11-I (2' OMe3+S) on (A, B) the weight of the liver (A) and serum AST and ALT levels (B) in mice.
(C, D) Body weight (C) and serum h-Alb profiles in HBV-infected human liver chimeric mice exposed to repeated treatment with of LNP-siRNA DOCK11-I (2' OMe3+S).
Data are presented as the mean (SD) (n = 3) and analyzed using the Mann–Whitney U-test. *P < 0.05, ***P < 0.001. NS, not significant.

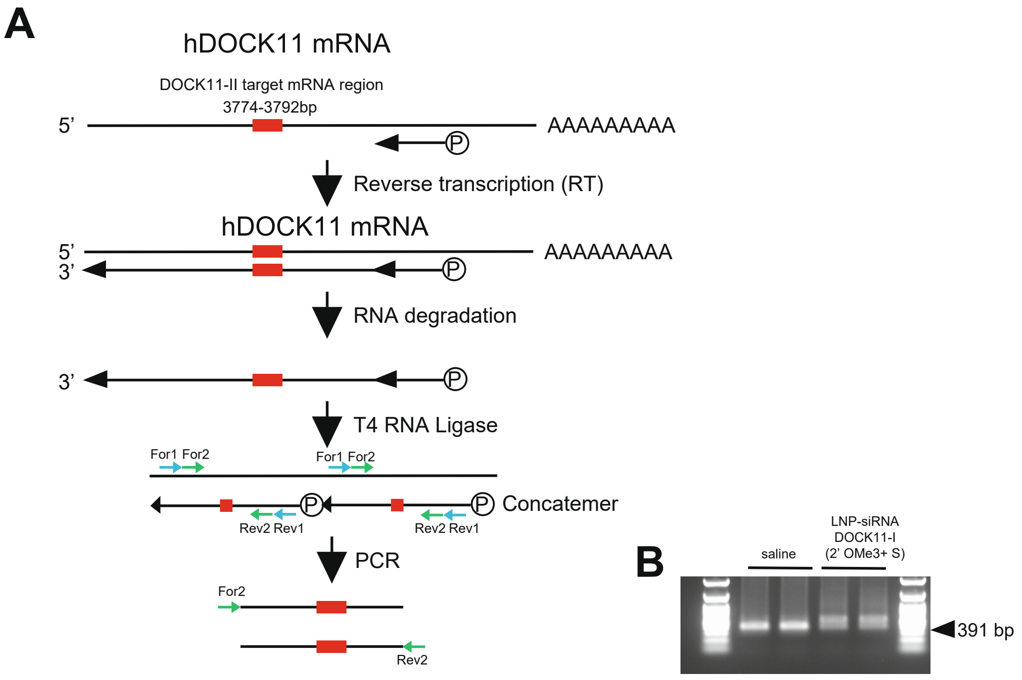

Figure S10. Cleavage efficiency of LNP-siRNA DOCK11-I (2' OMe3+S) against the target sequence of human DOCK11 mRNA in the liver tissue of HBV-infected human liver chimeric mice.
(A) Schematic of evaluating the cleavage efficiency of LNP-siRNA DOCK11-I (2' OMe3+S) against the target sequence of human DOCK11 mRNA.
(B) Cleavage efficiency of human DOCK11 target mRNA in the liver tissues of human liver chimeric mice chronically infected with HBV after repeated administration of Saline and LNP-siRNA DOCK11-I (2' OMe3+S).

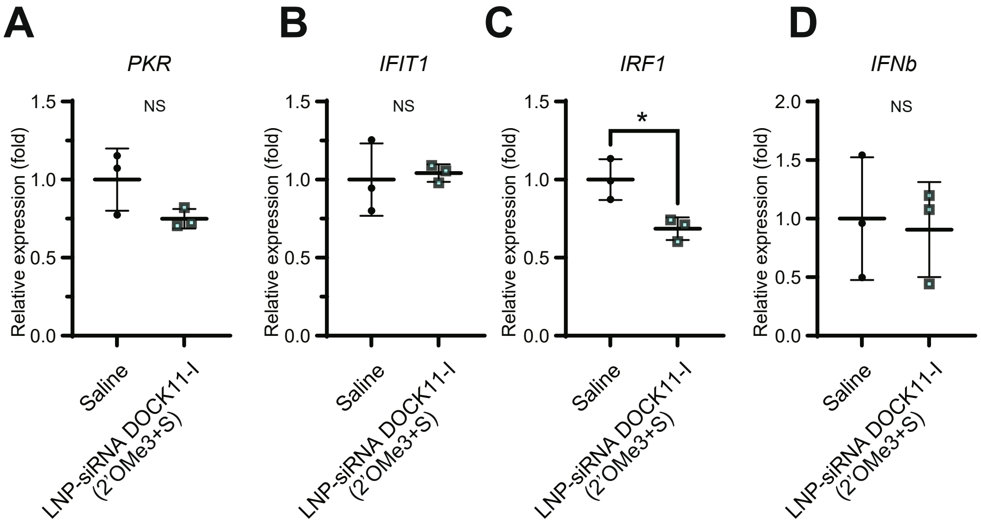

Figure S11. Effect of LNP-siRNA DOCK11-I (2' OMe3+S) on IFN-stimulated genes (ISGs) in HBV-infected human liver chimeric mice.
(A-D) Comparison of gene expression levels of PKR (A), IFIT1 (B), IRF1 (C), and IFNb (D) in human liver chimeric mice chronically infected with HBV after repeated administration of Saline and LNP-siRNA DOCK11-I (2' OMe3+S). Data are presented as mean (SD) (n = 3) and analyzed using the Mann–Whitney U-test. *$P < 0.05$. NS, not significant.
