# Supplementary material for: Lipid nanoparticle-encapsulated DOCK11-siRNA efficiently reduces hepatitis B virus cccDNA level in infected mice
Source: Mol Ther Methods Clin Dev. 2024 Jun 24;32(3):101289. doi: 10.1016/j.omtm.2024.101289 (PMC11300937; doi:10.1016/j.omtm.2024.101289)
Supplement: Document S2. Article plus supplemental information [file mmc2.pdf]

Molecular Therapy
Methods & Clinical Development
Original Article

Lipid nanoparticle-encapsulated DOCK11-siRNA efficiently reduces hepatitis B virus cccDNA level in infected mice

Hikari Okada,[1] Takeharu Sakamoto,[2] Kouki Nio,[3] Yingyi Li,[3] Kazuyuki Kuroki,[3] Saiho Sugimoto,[3] Tetsuro Shimakami,[3] Nobuhide Doi,[4] Masao Honda,[3] Motoharu Seiki,[3] Shuichi Kaneko,[1] and Taro Yamashita[3]

[1]Information-Based Medicine Development, Graduate School of Medical Sciences, Kanazawa University, Ishikawa, Japan; [2]Department of Cancer Biology, Institute of Biomedical Science, Kansai Medical University, Osaka, Japan; [3]Department of Gastroenterology, Kanazawa University Graduate School of Medical Science, Ishikawa, Japan; [4]Department of Biosciences and Informatics, Faculty of Science and Technology, Keio University, Kanagawa, Japan

The hepatitis B virus (HBV) infects many people worldwide. As HBV infection frequently leads to liver fibrosis and carcinogenesis, developing anti-HBV therapeutic drugs is urgent. Therapeutic drugs for preventing covalently closed circular DNA (cccDNA) production, which can eliminate HBV infection, are unavailable. The host factor dedicator of cytokinesis 11 (DOCK11) is involved in the synthesis and maintenance of HBV cccDNA *in vitro*. However, the effectiveness of DOCK11 as a target for the *in vivo* elimination of HBV cccDNA remains unclear. In this study, we assess whether DOCK11 inhibitors suppress HBV cccDNA production in mouse models of HBV infection. The tocopherol-conjugate hetero- gapmer, a DNA/RNA duplex of gapmer/complementary RNA targeting the DOCK11 sequence, partially reduces the expression of *DOCK11*, but not that of HBV cccDNA, in the livers of HBV-infected human hepatocyte chimeric mice, along with weight loss and decreased serum human albumin levels. Lipid nanoparticle-encapsulated chemically modified siRNAs specific for *DOCK11* suppress *DOCK11* expression and decrease HBV cccDNA levels without adverse effects in the mice. Therefore, nucleic acid-based drugs targeting DOCK11 in hepatocytes are potentially effective anti-HBV therapeutics that can reduce HBV cccDNA levels *in vivo*.

INTRODUCTION

Currently, more than 292 million people are affected by hepatitis B virus (HBV)-induced chronic hepatitis worldwide. Chronic hepatitis results in high rates of liver fibrosis, cirrhosis, and liver carcinogenesis, leading to more than 700,000 deaths annually.[1,2] This infection is associated with an intense rate of tumor growth after carcinogenesis.[3] HBV invades hepatocytes via interactions with heparan sulfate proteoglycan, sodium taurocholate cotransporting polypeptide (NTCP), and epidermal growth factor receptor (EGFR). HBV enters cells via the fusion of the viral envelope and the vesicle membrane followed by the nucleocapsid release into the cytoplasm. After the nuclear translocation, its incomplete double-stranded DNA (relaxed circular DNA [rcDNA]) genome undergoes DNA repair to form double-stranded covalently closed circular DNA (cccDNA), which is stably maintained in the nucleus and serves as a template for viral replication and antigen production, leading to persistent HBV infection. Pregenomic RNA, generated from cccDNA by the reverse transcriptase activity of polymerase in the HBV nucleocapsid, is reused to create rcDNA.[4] To date, several reverse transcriptase inhibitors, such as entecavir, have been developed and clinically applied. Recently, the development of the HBV vaccine has suppressed the onset of new infections.[5] Existing drugs can reduce HBV; however, they are not effective in eliminating cccDNA and the risk of virus recurrence.[6] Many drug development efforts are underway to target DNA repair mechanisms and prevent the production of cccDNA from HBV rcDNA. One of the most powerful tools in drug discovery is genome editing using the CRISPR-Cas9 system. This system is capable of directly deleting cccDNA in the genome, effectively preventing the synthesis of HBV-related genes. However, since DNA mutagenic agents that cause cccDNA formation defects target both virus and host DNA, concerns regarding side effects when administered over a long period remain. Therefore, targeting HBV cccDNA by developing new therapeutic drugs is crucial.[7]

Previous single-cell gene expression analyses identified the dedicator of cytokinesis 11 (DOCK11) as a host factor in HBV-infected hepatoma cells, which is involved in the maintenance of cccDNA.[8] DOCK11 is among the 12 DOCK family members. It functions as a guanine nucleotide exchange factor (GEF) and activates cell division cycle 42 (CDC42).[9,10] In cell culture experiments with HBV infection, DOCK11 showed GEF-mediated activation of CDC42 and ADP-ribosylation factor 1, further promoting viral replication by facilitating

Received 25 December 2023; accepted 21 June 2024;
https://doi.org/10.1016/j.omtm.2024.101289.

Correspondence: Hikari Okada, Information-Based Medicine Development, Graduate School of Medical Sciences, Kanazawa University, Ishikawa, Japan.
E-mail: okada0922@staff.kanazawa-u.ac.jp
Correspondence: Takeharu Sakamoto, Department of Cancer Biology, Institute of Biomedical Science, Kansai Medical University, Osaka, Japan.
E-mail: sakamott@hirakata.kmu.ac.jp

© 2024 The Authors. Published by Elsevier Inc. on behalf of The American Society of Gene and Cell Therapy.
This is an open access article under the CC BY license (http://creativecommons.org/licenses/by/4.0/).

infection from HBV virion entry to nuclear translocation. DOCK11 increases the efficiency of HBV infection by forming a complex with AGAP2 and facilitating the translocation of HBV from the Golgi apparatus to the nucleus.[11] The CDC42 activity-inhibitor peptide DCS8-42A, which acts at the DOCK homology region domain 2 of DOCK11, suppresses HBV replication *in vitro* and *ex vivo*. However, the administration of peptide DCS8-42A weakly affects HBV replication in HBV-infected human liver chimeric mice.[12] A CDC42-independent mechanism is potentially associated with reduced HBV cccDNA through suppressed DOCK11 expression but needs further validation.

Overexpressed DOCK11 promotes the synthesis of cccDNA from HBV rcDNA in HBV-infected NTCP-overexpressing HepG2 cells. DOCK11 is usually localized in the cytoplasm; however, it is translocated into the nucleus via EGF/EGFR signaling. Nuclear DOCK11 can repair UV-induced DNA damage through the ataxia telangiectasia and Rad3-related/checkpoint kinase 1 pathway.[12] Image analysis using super-resolution microscopy revealed the expression dynamics of DOCK11 in the nucleus but it did not colocalize with H3K4me3 and RNA polymerase II. DOCK11 can promote cccDNA transcriptional activation via the heterochromatin component HP1α.[13] The effects of DOCK11 on the HBV replication-related genes differ between the cytoplasm and nucleus, suggesting the role of DOCK11 in maintaining HBV replication; however, therapeutic effects of DOCK11-targeting drugs on the suppression of *in vivo* HBV replication remain unexplored.

In this study, we have performed preclinical analyses of nucleic acid modalities targeting *DOCK11* expression to investigate whether DOCK11 suppression reduces the abundance of HBV cccDNA in adeno-associated virus type 8 (AAV8)-HBV1.3mer-infected mice and HBV-infected human liver chimeric mice alleviating chronic infections.

RESULTS

DOCK11-targeting gapmers suppress the expression of DOCK11 and HBV cccDNA *in vitro*

To develop a nucleic acid-based medicine targeting DOCK11, we initially searched for the target mRNA sequence of DOCK11, which is conserved in mice and humans but exhibits low homology with other members of the DOCK family. In the human DOCK11 cDNA (6,219 bp) (NCBI reference: NM_144658.3), the sequence (3,369–3,819 bp) encoding the linker site of DOCK11 was selected for further development of nucleic acid drugs (Figure 1A). We chose single-stranded DNA antisense locked nucleic acid (LNA) gapmers as the modality of nucleic acid-based therapeutic strategy targeting DOCK11. Three types of gapmers were generated, which target three specific human sequences of DOCK11 mRNA located between 3,369 and 3,819 bp: #1 (3,778–3,792 bp), #2 (3,580–3,595 bp), and #3 (3,618–3,633 bp). Human hepatocellular carcinoma cell line Huh7 and mouse primary hepatocytes were transfected using these gapmers, and the inhibition of *DOCK11* expression was evaluated 48 h after transfection. In Huh7 cells, all gapmers reduced the DOCK11 mRNA levels in a concentration-dependent manner (Figure 1B). In primary mouse hepatocytes, only DOCK11#1 reduced DOCK11 mRNA levels in a concentration-dependent manner (Figure 1C). Considering the highest homology between the sequences of DOCK11 and DOCK9 mRNAs,[14] we examined the specificity of DOCK11 gapmers using human umbilical vein endothelial cells (HUVECs) expressing both DOCK11 and DOCK9. The gapmer DOCK11#1 specifically suppressed the expression of *DOCK11* rather than *DOCK9*, whereas the other two gapmers downregulated both DOCK11 and DOCK9 (Figures S1A and S1B). We further investigated the gapmer DOCK11#1 exhibiting suppressive effects on *DOCK11* expression in both human and mouse cells without altering *DOCK9* expression. Further optimization of gapmer lengths for DOCK11 expression suppression revealed that the 13 bp gapmer DOCK11#1 lost its suppressive effect on *DOCK11*, while the 15 and 17 bp gapmer DOCK11#1 exhibited comparable suppressive effects on DOCK11 expression in Huh7 cells (Figures 1D and 1E). Gapmers are internalized into cells by endocytosis rather than passive transport; the efficiency of cellular uptake decreases with increasing length of the gapmer sequence.[15] Hence, the 15 bp gapmer DOCK11#1 was chosen for further analyses. We examined whether gapmer DOCK11#1-mediated suppression of DOCK11 reduces HBV cccDNA levels *in vitro*. In human hepatocarcinoma HepG2.2.15 cells with stable HBV expression and replication, the DOCK11#1 gapmer decreased both DOCK11 mRNA and HBV cccDNA levels (Figures 1F and 1G).[16] Therefore, targeting DOCK11 using gapmers is considered effective in eliminating HBV cccDNA *in vitro*.

The DOCK11-targeting gapmer transiently decreases *DOCK11* expression but is insufficient to eliminate HBV cccDNA *in vivo*

Subsequently, we investigated the inhibitory efficacy of the gapmer DOCK11#1 *in vivo*. After injecting 10 mg/kg gapmer DOCK11#1 solution into the tail vein of wild-type C57BL/6J mice, *Dock11* expression was suppressed in the liver tissue by approximately 40% on day 2; however, it was recovered on day 5 (Figures 2A–2C), indicating that gapmer DOCK11#1 downregulates *Dock11* in the liver of mice for a short period. Alanine aminotransferase (ALT) and aspartate aminotransferase (AST) values were the highest, followed by those of the gapmer DOCK11#1 group. These ALT/AST values returned to normal levels in each group on the fifth day of administration (Figures S2A and S2B). Furthermore, while analyzing whether continuous administration of the DOCK11#1 can suppress HBV replication, HBV replication was assessed in HBV-infected human liver chimeric mice, which was generated by replacing approximately 70% of the mouse hepatocytes of uPA/SCID immunodeficient mice with human hepatocytes. To create a mouse model of chronic HBV infection, human liver chimeric mice were infected with HBV genotype C particles (1×10^5 copies/mouse) and maintained for 8 weeks. Next, 10 mg/kg gapmer scramble or DOCK11#1 was injected into the tail vein of HBV-infected human liver chimeric mice every 4 days (Figure 2D). However, no changes in *DOCK11* mRNA expression, HBV DNA, or HBV cccDNA levels were detected in

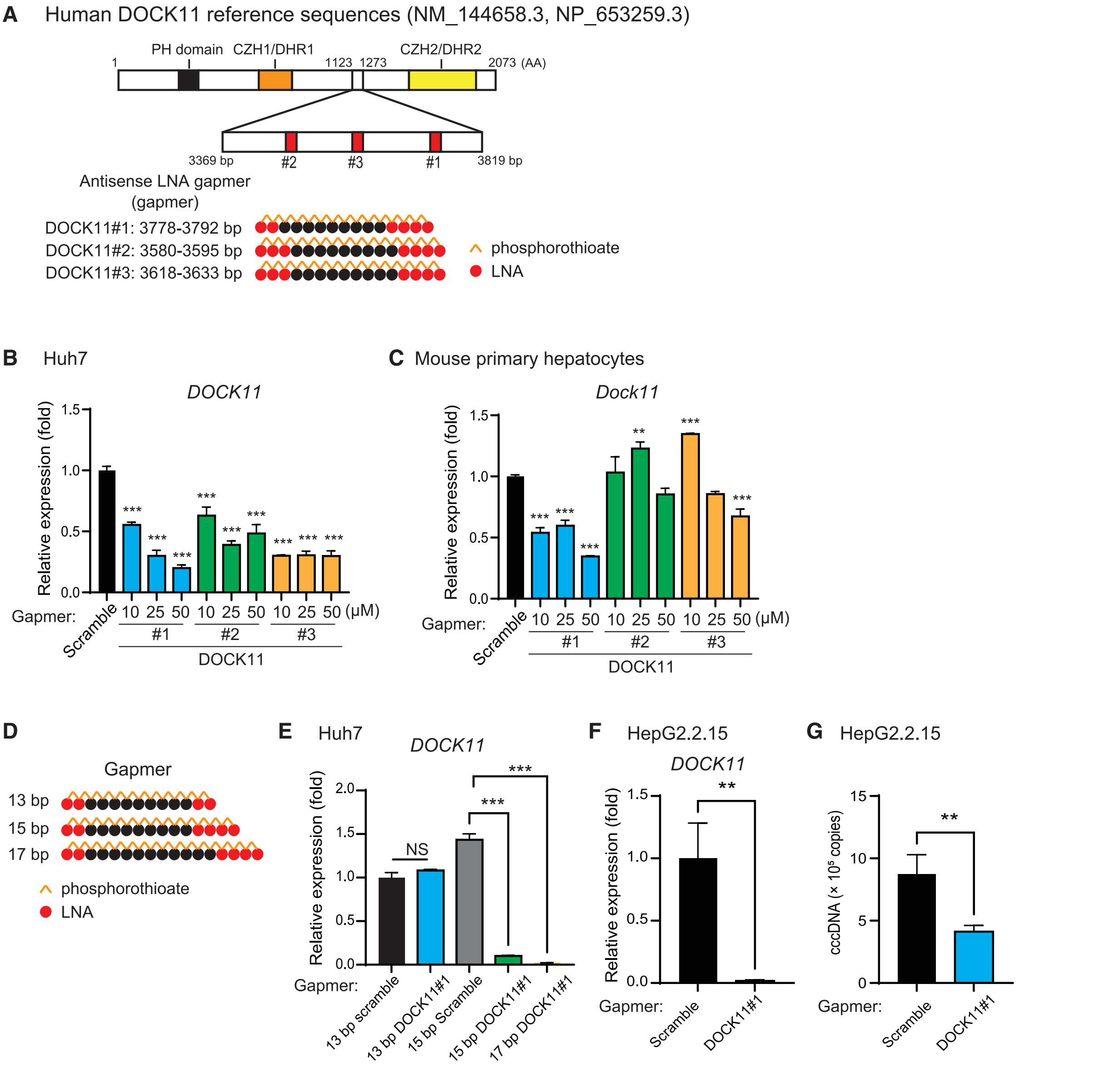

Figure 1. DOCK11-targeting gapmers suppress DOCK11 expression and HBV cccDNA *in vitro*

(A) Schematic illustration of the full-length human DOCK11 and the gapmer constructs. (B and C) *DOCK11* mRNA expression in Huh7 cells (B) and mouse primary hepatocytes (C) 48 h after gapmer treatment. (D) Schematic illustration of 13, 15, and 17 bp DOCK11#1 gapmers. (E) *DOCK11* mRNA expression in Huh7 cells transfected with gapmer scramble and DOCK11#1. (F and G) *DOCK11* mRNA expression (F) and HBV cccDNA copy number (G) in Hep2.2.15 cells transfected with gapmer scramble and DOCK11#1. In (B), (C), and (E–G), data are presented as the mean (SD) ($N = 3$) and analyzed using the one-way ANOVA. *$p < 0.05$, **$p < 0.01$, ***$p < 0.001$; NS, not significant.

the livers of gapmer DOCK11-administrated mice compared with those of scrambled gapmer-administered mice (Figures 2E–2G). Administration of the gapmer DOCK11#1 did not decrease blood HBV DNA and HBsAg levels (Figures S3A and S3B). Therefore, gapmer DOCK11#1 administration suppressed DOCK11 expression within 2 days; however, the duration of suppression was short. Therefore, nucleic acid administration as a gapmer was insufficient in eliminating cccDNA from HBV-infected human liver chimeric mice.

The hetero-gapmer targeting DOCK11 shows an extended duration of DOCK11 suppression and decreases the HBV cccDNA level *in vivo*

To improve the delivery efficacy into the liver and anti-DOCK11 effects of gapmer DOCK11#1, a heterologous nucleic acid format of the gapmer modified with α-tocopherol-triethylene glycol (TEG)[17] was explored. We synthesized complementary RNA (cRNA) using the nucleic acid sequence of gapmer DOCK11#1 and modified its 5′ side using α-tocopherol-TEG.[18]

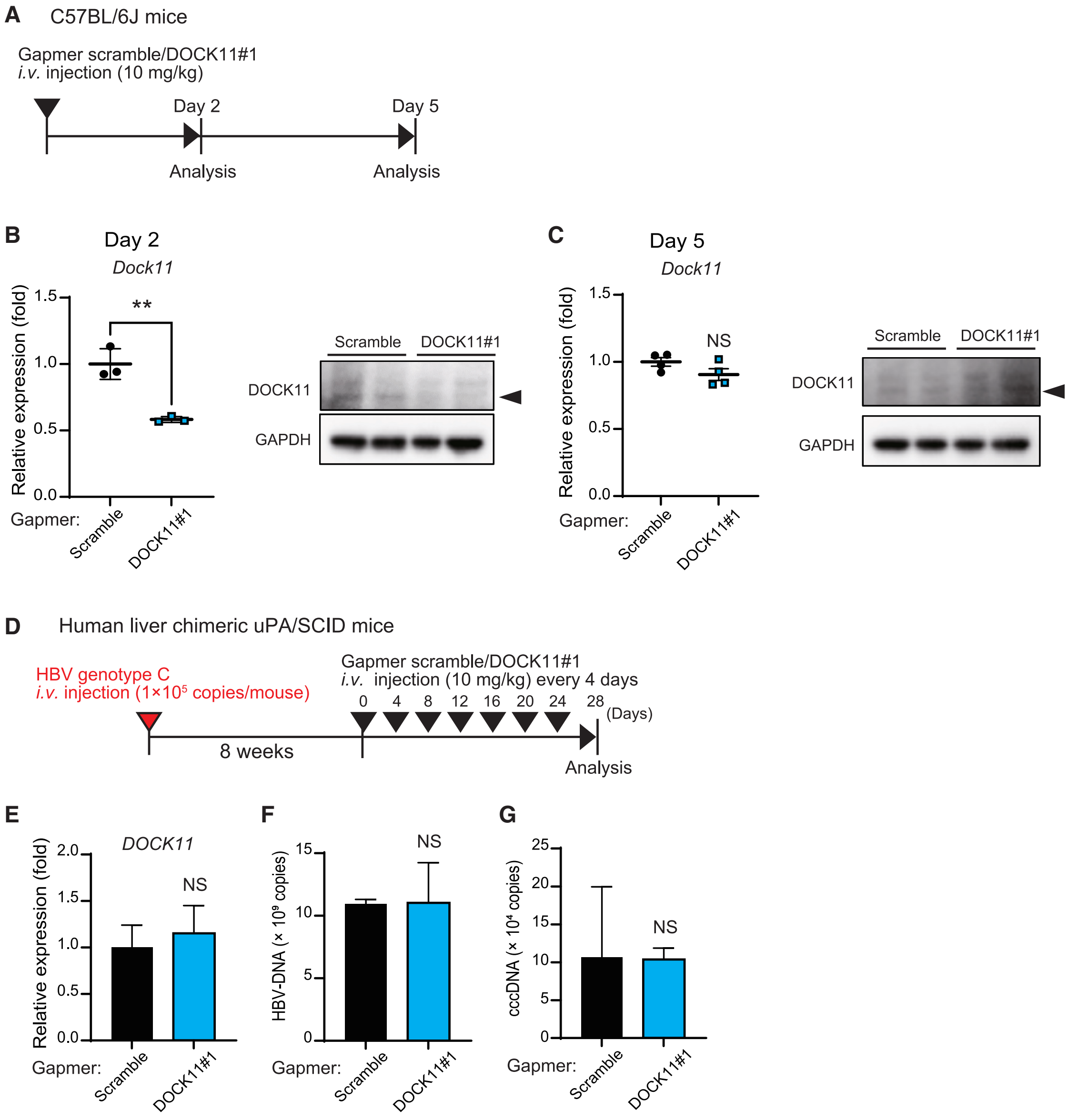

Figure 2. The DOCK11-targeting gapmer transiently suppresses the expression of DOCK11 but is insufficient to eliminate HBV cccDNA *in vivo*

(A) The schedule for gapmer administration in animals. (B and C) Expression levels of *DOCK11* mRNA ($N = 3$) and protein ($N = 2$) in the liver at 2 days (B) and 5 days ($N = 4$) (C) after injecting gapmers. (D) Experimental schedule for administration of gapmer scramble and DOCK11#1 in human liver chimeric mice with HBV chronic infections. (E–G) *DOCK11* mRNA expression (E), and copy numbers of HBV DNA (F) and cccDNA (G) in the liver of HBV-infected human liver chimeric mice treated with gapmers ($N = 2$). In (B), (C), and (E–G), data are presented as the mean (SD) and analyzed using the Mann-Whitney U test. $^{**}p < 0.01$; NS, not significant.

Then, gapmer DOCK11#1 and cRNA-tocopherol were combined by heating to generate a hetero-gapmer (Figure 3A). The effect of this newly created hetero-gapmer DOCK11#1 on *DOCK11* expression in the liver was validated using wild-type C57BL/6J mice (Figure 3B). The hetero-gapmer DOCK11#1 (3 mg/kg) strikingly suppressed *DOCK11* expression in the liver even 5 days after injection (Figure 3C), indicating a longer period of inhibitory effect compared with that of the single-chain gapmer DOCK11#1.

Next, we investigated the hetero-gapmer DOCK11-induced suppression of HBV replication and HBV cccDNA levels in the AAV8-HBV1.3mer infection model, which was generated by infecting wild-type C57BL/6J mice using 1×10^{10} copies/mouse of AAV8-HBV1.3mer viral particles leading to the peak of HBV replication approximately 30 days after infection.[19] The hetero-gapmer scramble and DOCK11#1 were separately injected 10 and 25 days after AAV8-HBV1.3mer infection for analyzing the early and chronic phases of infection (Figure 3D). On day 15 (the early phase

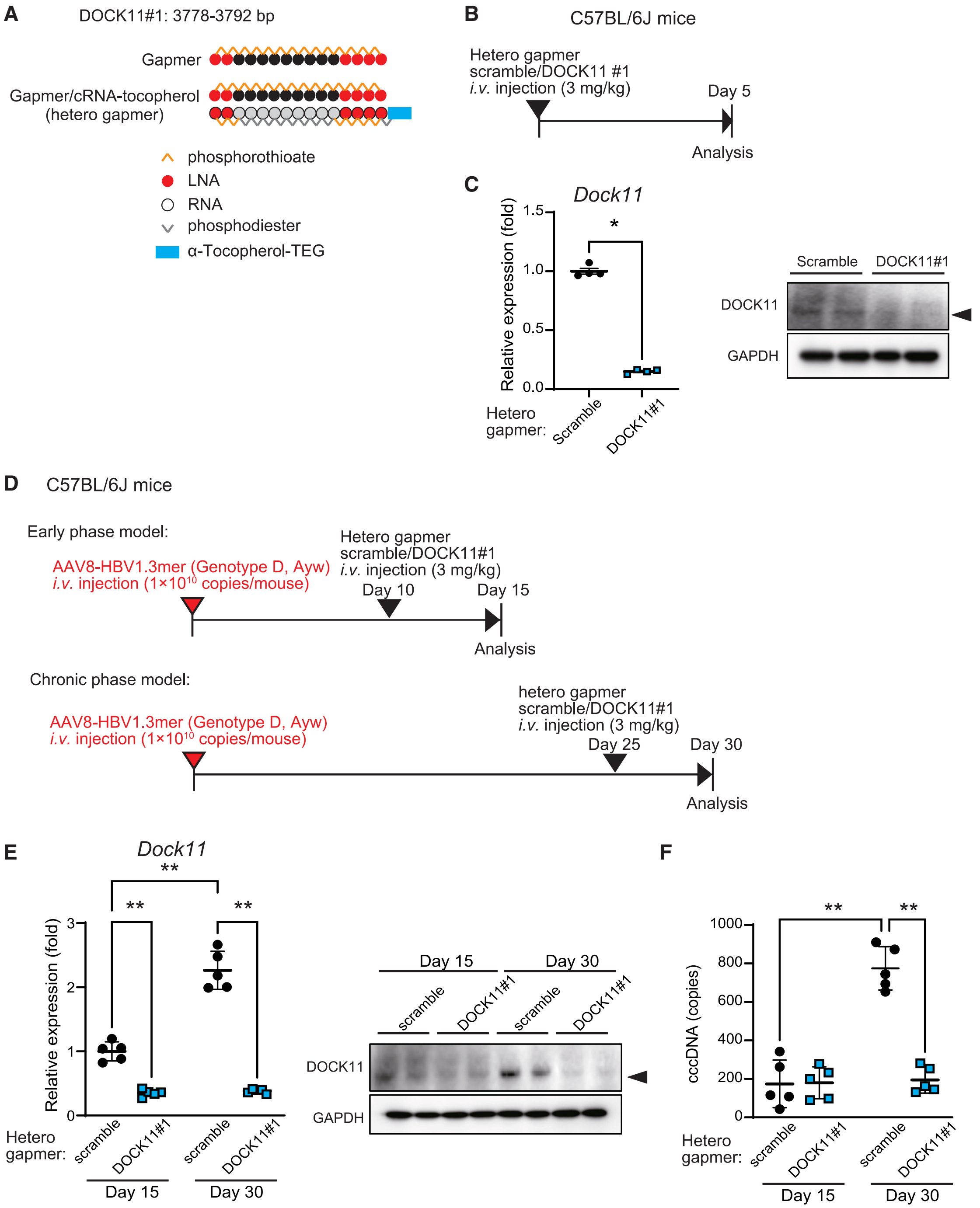

Figure 3. The DOCK11-targeting hetero-gapmer exhibits stable DOCK11-suppressive effects and decreases HBV cccDNA levels *in vivo*
(A) Schematic presentation demonstrating hetero-gapmer constructs. (B) Schedule for nucleic acid administration in animals. (C) Expression of *Dock11* mRNA ($N = 4$) and protein in the liver of C57BL6/J mice 5 days after administration of hetero-gapmer. (D) Schedule for the administration of hetero-gapmers in AAV8-HBV1.3mer virus-infected C57BL/6J mice (early-phase and chronic-phase models). (E and F) Expression of *Dock11* mRNA, protein (E) and cccDNA copy number (F) in the liver of hetero-gapmer-treated mice on days 15 and 30 ($N = 5$). In (C), (E), and (F), data are presented as the mean (SD) and analyzed using the Mann-Whitney U test. $*p < 0.05$, $**p < 0.01$.

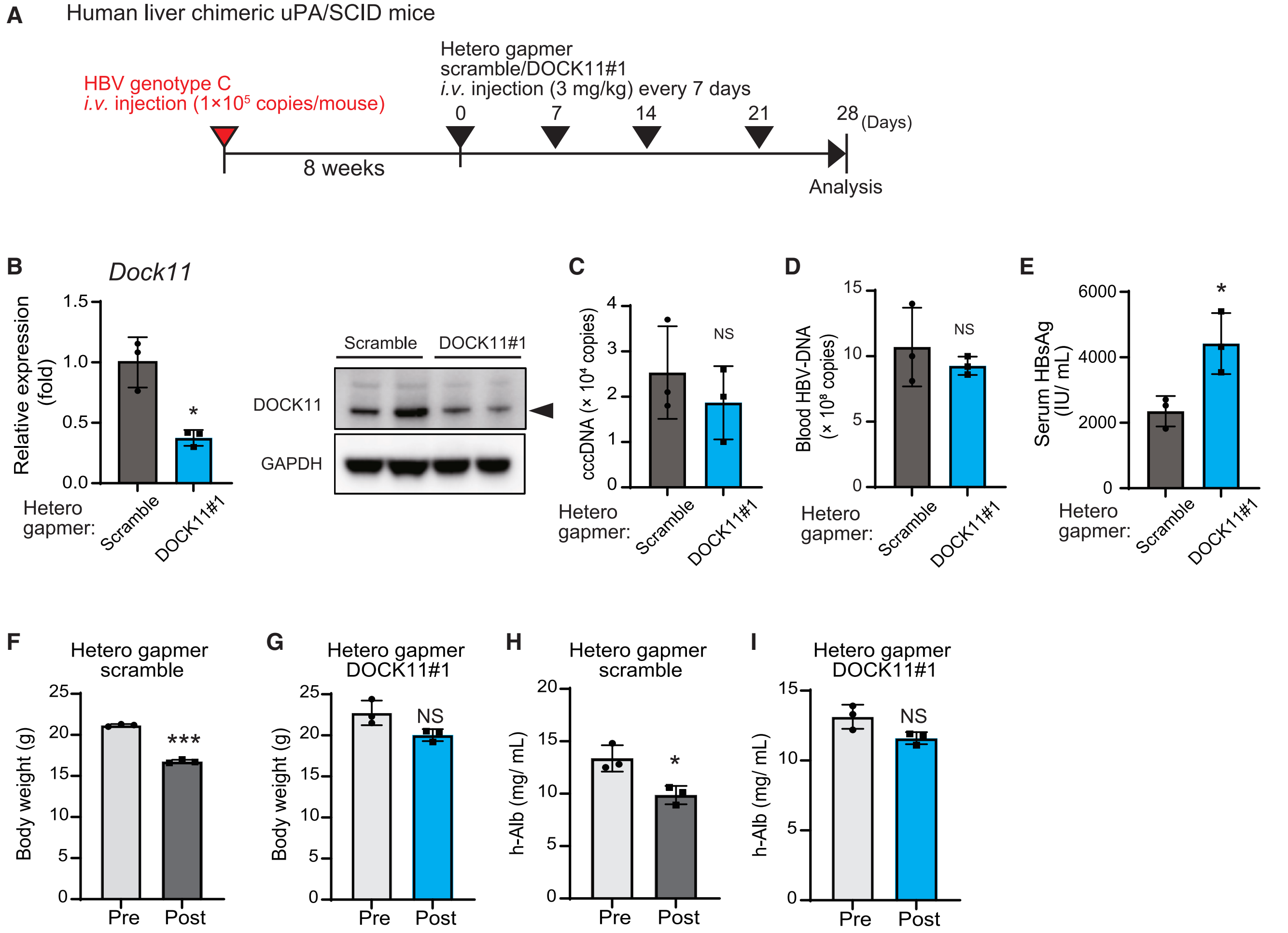

Figure 4. Hetero-gapmers are hepatotoxic in HBV-infected human liver chimeric mice

(A) Schedule for the administration of hetero-gapmers in HBV-infected human liver chimeric mice. (B–E) Expression of hepatic *DOCK11* mRNA, protein (B), hepatic HBV cccDNA (C), serum HBV-DNA (D), and serum HBsAg (E) in human liver chimeric mouse models of HBV chronic infection, after hetero-gapmer administration. (F–I) Changes in body weights (F and G) and h-Alb levels (H and I) of HBV-infected human liver chimeric mouse after administration of the hetero- gapmer scramble (F and H) and DOCK11#1 (G and I). In (B)–(I), data are presented as the mean (SD) ($N = 3$) and analyzed using the two-sided unpaired t test with Welch's correction. $^{*}p < 0.05$, $^{**}p < 0.01$, $^{***}p < 0.001$; NS, not significant.

of HBV infection), administration of the hetero-gapmer DOCK11#1 reduced expression of DOCK11 but not HBV cccDNA levels (day 15, Figures 3E and 3F). On day 30 (the chronic phase of HBV infection), *DOCK11* expression and cccDNA levels were increased in the liver of AAV8-HBV1.3mer-infected mice treated with hetero-gapmer scramble, whereas, hetero-gapmer DOCK11#1 significantly suppressed *DOCK11* expression and HBV cccDNA levels (Figures 3E and 3F). When hetero-gapmer DOCK11#1 was administered to HBV acute/chronic infection model mice, no increase in AST/ALT levels was observed compared with scramble (Figures S4C and S4D). Administration in wild-type mice showed similar results (Figures S4A and S4B). These results indicate that a single dose of the hetero-gapmer DOCK11#1 potentially suppresses HBV replication caused by AAV8-HBV 1.3mer infection during the chronic phase of infection.

Hetero-gapmers are hepatotoxic in HBV-infected human liver chimeric mice

Furthermore, we examined whether the hetero-gapmer DOCK11#1 can suppress replication and cccDNA levels of HBV in human liver chimeric mouse models of HBV infection. Hetero-gapmer scramble and DOCK11#1 were injected every 7 days into human liver chimeric mice with HBV infection for 10 weeks, and the mouse livers were analyzed 28 days after the first injection (Figure 4A). In the liver, the hetero-gapmer DOCK11#1 inhibited Dock11 expression by approximately 60% but HBV cccDNA, blood HBV-DNA, and serum HBsAg levels remained unaltered in HBV-infected mice (Figures 4B–4E). Hetero-gapmers were toxic in HBV-infected human liver chimeric mice; it reduced the body weight and serum human albumin level and increased AST/ALT levels in mice (Figures 4F–4I, S5A, and S5B). The hetero-gapmer scramble showed more severe adverse

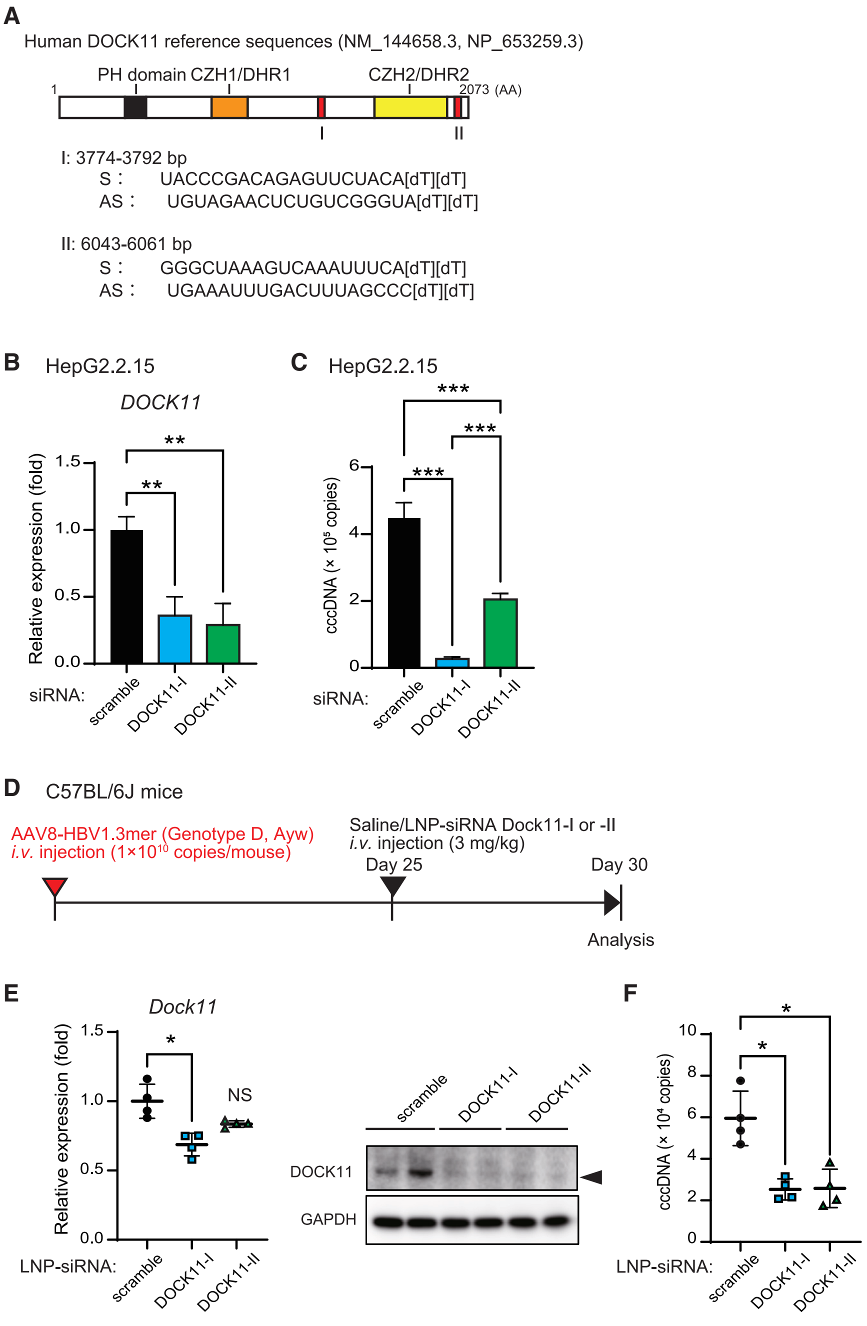

Figure 5. LNP-encapsulated siRNA targeting DOCK11 reduces HBV cccDNA *in vivo*

(A) Construction of siRNA against DOCK11. (B and C) The relative expression levels of *DOCK11* mRNA (B) and HBV cccDNA copy number (C) in Hep2.2.15 cells 48 h after siRNA transfection; data are presented as the mean (SD) (N = 3) and were analyzed using the one-way ANOVA. *p < 0.05, **p < 0.01, ***p < 0.001. (D) Schedule for the administration of LNP-siRNA in AAV8-HBV1.3mer-infected C57BL6/J mice. (E and F) The relative expression levels of *DOCK11* mRNA, protein (E) and HBV cccDNA copy number (F) in the livers of LNP-siRNA-treated mice. Data are presented as the mean (SD) (N = 4) and analyzed using the Mann-Whitney U test. *p < 0.05.

effects than the hetero-gapmer DOCK11 (Figures S5A and S5B). Thus, these hetero-gapmers are considered hepatotoxic and unsuitable for the *in vivo* therapy of HBV infection, particularly in HBV-infected human liver chimeric mouse models.

Lipid nanoparticle-encapsulated DOCK11-targeting siRNA decreases HBV cccDNA *in vivo*

To avoid *in vivo* toxicity, we changed the nucleic acid modality from a hetero-gapmer to siRNA[20] to target DOCK11. For the DOCK11-I sequence, a large region was determined from the full-length DOCK11 mRNA sequence and narrowed down to 15 bp for gapmer and 21 bp (3,774–3,792 bp) for siRNA. Next, we removed 3,369–3,819 bp from the human DOCK11 mRNA sequence and designated 6,043–6,061 bp as DOCK11-II, a sequence with low homology with other genes and suitable for siRNA construction (Figure 5A). These two siRNAs (siRNA DOCK11-I and -II) were used to transfect Hep2.2.15 cells and DOCK11 expression and HBV cccDNA levels were analyzed. Both siRNAs decreased *DOCK11* expression and HBV cccDNA levels in HepG2.2.15 cells (Figures 5B and 5C). After *in vitro* verification, siRNA was encapsulated using lipid nanoparticles (LNPs) to generate the LNP-siRNA formulation. The composition of the LNPs was equivalent to that of patisiran.[21] LNP-siRNA DOCK11-I and -II were singly injected into AAV8-HBV1.3mer-infected model mice (Figure 5D). LNP-siRNA DOCK11-I and -II decreased Dock11 expression and HBV cccDNA levels in the liver of AAV8-HBV1.3mer-infected mice (Figures 5E and 5F). Hence, the LNP-siRNA formula is considered potentially applicable in the further development of DOCK11 targeting nucleic acid medicine.

Enhanced *in vitro* efficacy of chemically modified DOCK11-targeting siRNA

To enhance the efficacy, specificity, and stability of the siRNA, chemical modifications were introduced to the siRNAs DOCK11-I and -II. First, 2′-O-methylation (2′OMe)[22] of 1, 2, 3, and 4 bases at both ends of the DOCK11-I and -II siRNA sequences were applied (Figure 6A). HepG2.2.15 cells were transfected using these siRNAs and, subsequently, DOCK11 expression and HBV cccDNA levels were analyzed 72 h after transfection; DOCK11-I harboring 3 and 4 bases with

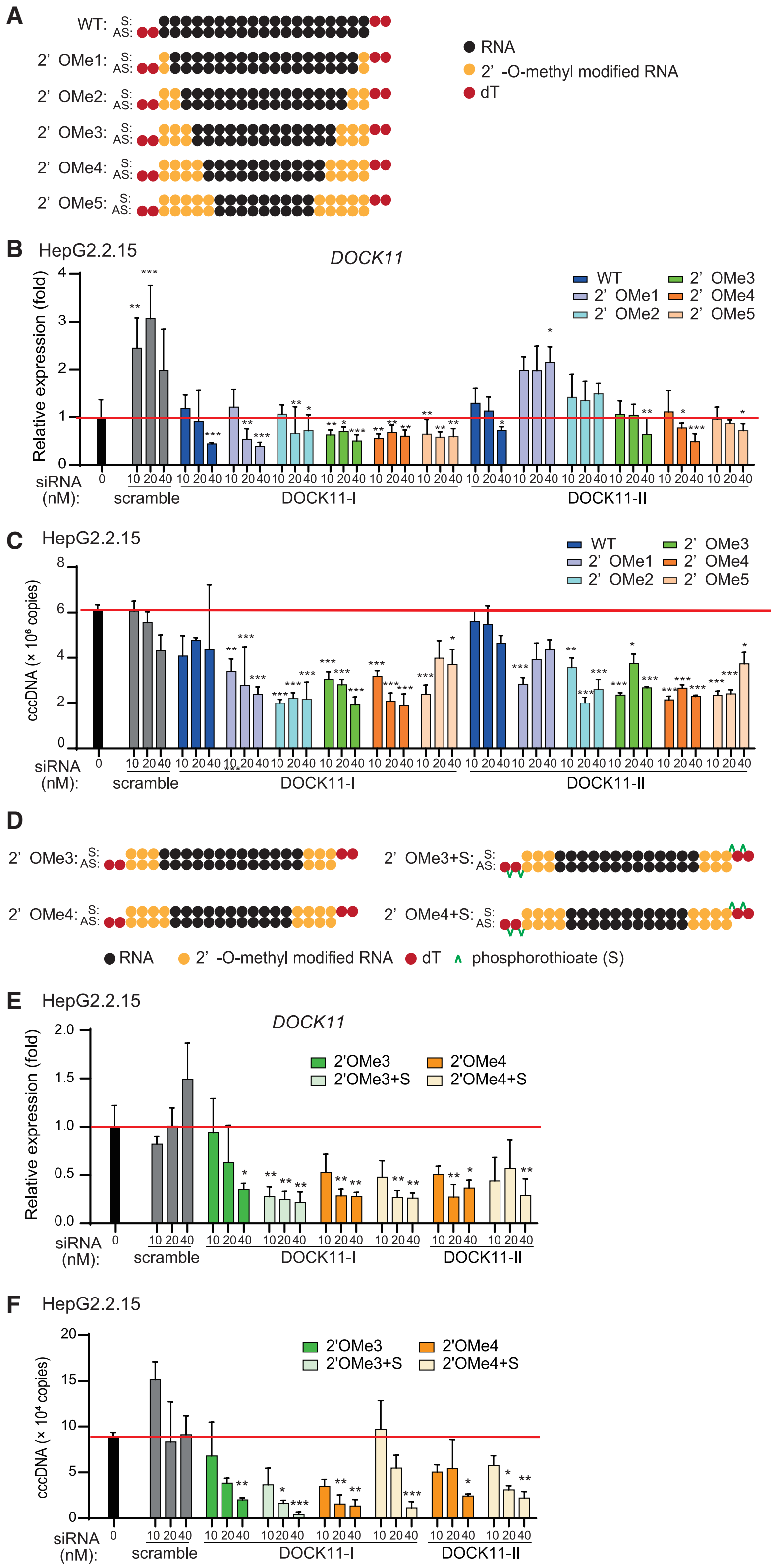

Figure 6. Chemically modified DOCK11-targeting siRNA exhibits enhanced efficacy for gene suppression and HBV cccDNA elimination *in vitro*

(A) Schematic representation of the chemically modified siRNAs indicating positions of 2′-O-methylation (2′OMe). (B and C) The relative expression levels of *DOCK11* mRNA (B) and HBV cccDNA copy number (C) in Hep2.2.15 cells after transfection of 2′-OMe siRNA (N = 3). (D) Schematic representation of the chemically modified siRNAs indicating positions of 2′OMe and phosphorothioate (S) modifications. (E and F) The relative expression levels of *DOCK11* mRNA (E) and HBV cccDNA copy number (F) in HepG2.2.15 cells 72 h after transfection of siRNA modified with 2′OME and phosphorothioate. The red line, indicating the data obtained from the untreated group, is considered for comparative analyses. In (B), (C), (E), and (F), data are presented as the mean (SD) (N = 3) and analyzed using the one-way ANOVA. *$p < 0.05$, **$p < 0.01$, ***$p < 0.001$.

2′OMe modifications and DOCK11-II siRNA harboring 4 bases with 2′OMe modifications efficiently and reproducibly suppressed DOCK11 mRNA and HBV cccDNA levels (Figures 6B and 6C). Subsequently, siRNA DOCK11 with 2′OMe modifications was further phosphorothioated[23] at the overhangs (Figure 6D). The chemically modified siRNA DOCK11 was used to transfect HepG2.2.15 cells and DOCK11 expression; analysis of HBV cccDNA level that, among chemically modified siRNAs, siRNA DOCK11-I with 3 base 2′OMe modifications and phosphorothioated overhangs exhibited the highest inhibitory efficacy against DOCK11 and HBV cccDNA (Figures 6E and 6F). Hence, we further evaluated chemically modified siRNA DOCK11-I.

LNP-encapsulated chemically modified siRNA is more effective *in vivo* against HBV infection than other nucleic acid modalities in a chronic AAV8-HBV1.3mer infection model mouse

We verified whether DOCK11-I chemically modified siRNA was effective against HBV replication *in vivo*. Gapmer, unmodified siRNA, and chemically modified siRNA were wrapped in LNPs and administered as a single dose to AAV8-HBV1.3mer chronically infected model mice at 4 mg/kg (Figure 7A). Seven days after administration, in the group administered with LNP-encapsulated chemically modified siRNA, the expression of DOCK11 was suppressed compared with the three groups including saline (Figure 7B), and the amount of cccDNA was reduced (Figure 7C). Compared with the saline group, the ALT/AST values, which are markers of liver damage, did not change in the LNP chemically modified siRNA-treated group but increased in the LNP non-modified siRNA-treated group (Figures S6A and S6B). These results supported the high efficacy of LNP chemically modified siRNA compared with other nucleic acid modalities.

Reverse transcriptase inhibitors, such as entecavir, are the most common clinical drugs that inhibit HBV replication. Patients with HBV experience recurrence of viral replication if reverse transcriptase inhibitor doses are stopped; relapsed HBV infection is attributed to the cccDNA remaining in hepatocytes, which is the source of factors involved in the duplication of HBV particles.[24] Conceivably, in our AAV8-HBV1.3mer infection mouse models, entecavir suppressed HBV-DNA but not HBV cccDNA (Figures S8A–S8D). LNP-siRNA DOCK11-I prevented the increase in HBV cccDNA in a relapse mouse model of HBV infection associated with the discontinuation of entecavir treatment (Figures 7D–7G). Therefore, the LNP-siRNA preparation is potentially effective against HBV recurrence associated with mid-term discontinuation of entecavir doses, which is a major clinical problem.

LNP-encapsulated chemically modified siRNA targeting DOCK11 inhibits replication and cccDNA of HBV in infected human hepatocyte chimeric mice

Next, the effects of LNP-siRNA DOCK11-I were evaluated in HBV-infected human liver chimeric mouse models. LNP-siRNA DOCK11-I (2′OMe3+S) (4 mg/kg body weight) was intravenously injected into HBV-infected human liver chimeric mice every 7 days (Figure 8A). Repeated administration of the LNP-siRNA DOCK11-I (2′OMe3+S) suppressed *DOCK11* expression in the liver of HBV-infected human liver chimeric mice by approximately 80%, which indicated enhanced effectiveness over that of the previously used nucleic acid drugs (Figure 8B). Furthermore, LNP-siRNA DOCK11-I (2′OMe3+S) reduced HBV-DNA, HBsAg, and HBeAg levels in the blood by 60%, 20%, and 30%, respectively, compared with those recorded in the saline-treated counterparts (Figures 8C–8E). In addition, LNP-siRNA DOCK11-I (2′OMe3+S) reduced HBV-DNA and HBV cccDNA levels in the liver of HBV-infected human liver chimeric mice by more than 50% (Figures 8F–8H). Immunostaining analyses of liver tissues revealed reduced levels of HBsAg and HBcAg in LNP-siRNA DOCK11-I (2′OMe3+S)-treated mice than in the control mice (Figure 8I). Remarkably, LNP-siRNA DOCK11-I (2′OMe3+S) did not affect liver and body weight and serum human albumin levels; AST/ALT values were attenuated (Figures S9A–S9D). LNP-siRNA DOCK11-I (2′OMe3+S) could specifically cleave DOCK11 mRNA in liver tissues of HBV-infected human liver chimeric mice (Figures S10A and S10B) without triggering RNA-mediated innate immune activation (Figures S11A–S11D). Overall, the LNP-siRNA DOCK11-I suppressed HBV replication and HBV cccDNA levels *in vivo* without any adverse effects.

DISCUSSION

Despite the availability of many medicines that inhibit HBV replication, no drugs are available that can target HBV cccDNA.[25] Although there is an innovative genome editing technology that directly eliminates HBV cccDNA and inhibits HBV replication, it cannot be ignored that there may be permanent effects on host genes due to off-target effects and the induction of gene mutations.[26,27] We have demonstrated by inhibiting *DOCK11* expression using LNP-siRNA, which is used in clinical settings and can circumvent these issues, that the amount of HBV cccDNA during chronic HBV infection can be reduced. The LNP-siRNA targeting DOCK11 more effectively suppressed HBV cccDNA with no apparent adverse effects compared with gapmers and hetero-gapmers. We demonstrated the possibility that a DOCK11 expression inhibitor could suppress the synthesis of HBV cccDNA from rcDNA. Detailed reports on the binding mechanism between DOCK9 or DOCK10 and CDC42 exist[28–30] but not on DOCK11. It is currently unclear whether DOCK11 maintains HBV cccDNA through a CDC42-independent pathway or an unknown binding mechanism with CDC42. However, the remaining (approximately 50%) HBV cccDNA in LNP-siRNA DOCK11-treated HBV-infected human liver chimeric mouse models indicates that improvement of the LNP-siRNA DOCK11 and/or the combination of DOCK11 targeting therapy and other HBV inhibitors is necessary for the improved efficacy of HBV therapy.

In addition to siRNA, we explored the effectiveness of gapmers in the therapeutic targeting of HBV using cell lines and mouse models. Gapmers are antisense DNA that degrade target RNA by activating RNase H. RNase H activity promotes HBV replication by enhancing HBV-DNA synthesis.[31] However, owing to the low structural similarity of the host and viral RNase H molecules, gapmer-mediated activity

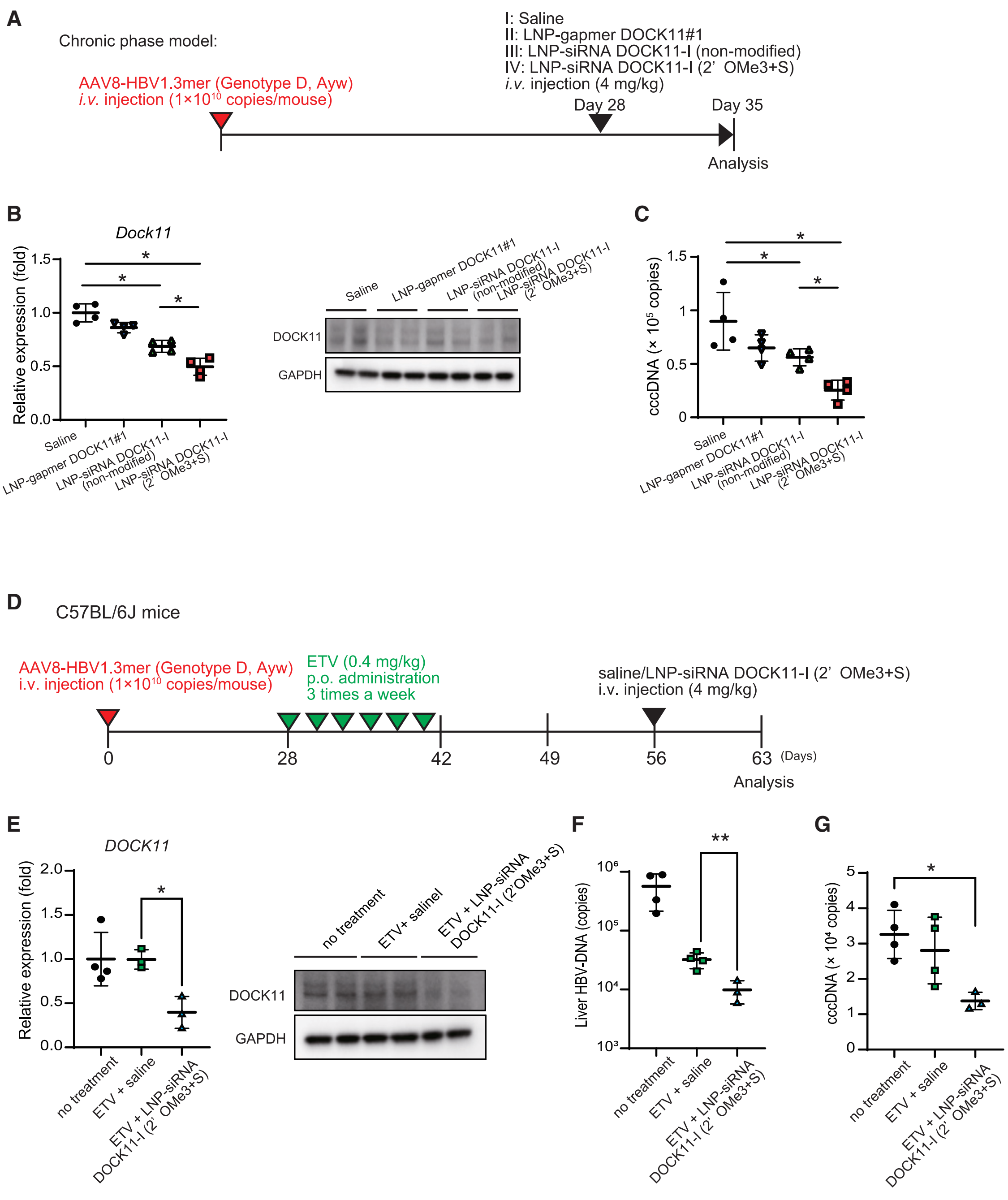

Figure 7. LNP chemically modified siRNA suppresses HBV cccDNA in AAV8-HBV1.3mer chronic infection model mice and HBV relapse model of HBV infection post-ETV discontinuation

(A) Experimental schedule for administering LNP-siRNA DOCK11-I (2′OMe3+S) to AAV8-HBV1.3mer chronically infected mice. (B and C) The relative expression of DOCK11 mRNA and protein (B) and copy numbers of HBV cccDNA (C) in the livers of mice treated with saline, LNP-gapmer DOCK11#1, LNP-siRNA DOCK11 (non-modified), and LNP-siRNA DOCK11 (2′OMe3+S). (D) Schedule for establishing the HBV relapse model with entecavir and administration of LNP-siRNA DOCK11 (2′OMe3+S). (E–H) The relative expressions of DOCK11 mRNA, protein (E), HBV DNA (F), and HBV cccDNA (G) in the liver of relapse mouse model of AAV8-HBV1.3mer HBV infection 7 days after a single administration of LNP-siRNA DOCK11-I (2′ OMe3+S). Data are presented as the mean (SD) ($N = 3$–4) and analyzed using the two-sided unpaired t test with Welch's correction. *$p < 0.05$, **$p < 0.01$.

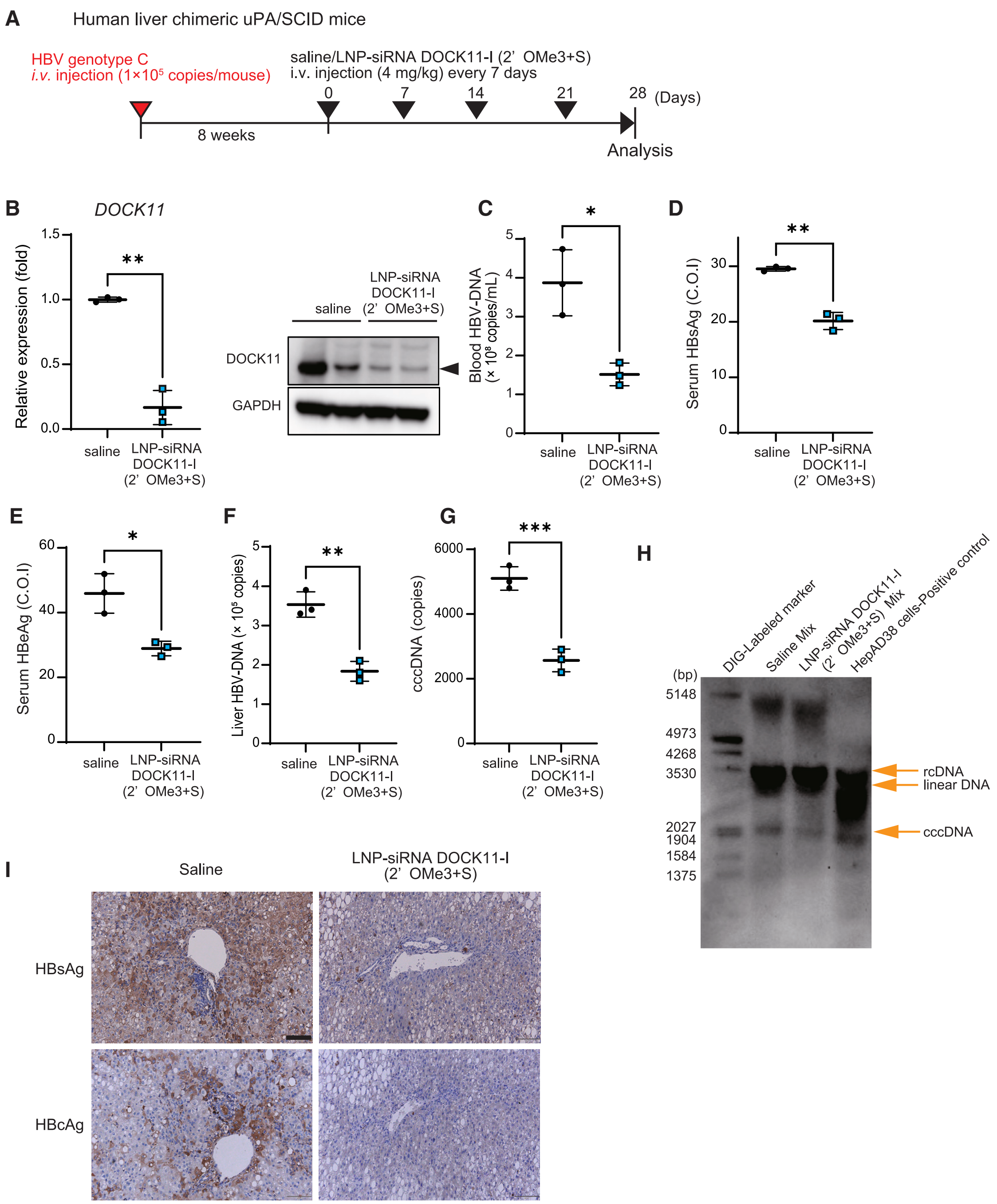

Figure 8. LNP-encapsulated chemically modified DOCK11-targeting siRNA reduces HBV replication and cccDNA in HBV-infected human hepatocyte chimeric mice

(A) Experimental schedule for administration of LNP-siRNA DOCK11-I (2′OMe3+S) in HBV-infected human liver chimeric mice. (B–G) The relative expression levels of *DOCK11* mRNA and protein in the liver (B), the copy number of blood HBV-DNA (C), serum HBsAG (D), and HBeAG (E) levels, and the copy numbers of HBV cccDNA (F) and HBV-DNA (G) in the liver of HBV-infected human liver chimeric mice after administering LNP-siRNA DOCK11-I (2′OMe3+S). (H) The amounts of rcDNA, linearDNA, and cccDNA in the liver tissues of human liver chimeric mice were analyzed by Southern blotting. (I) Immunohistochemical staining analyses of HBsAg and HBcAg in the liver of human liver chimeric mice. Scale bar, 100 μm. Data are presented as the mean (SD) (N = 3) and analyzed using the two-sided unpaired t test with Welch's correction. *$p < 0.05$, **$p < 0.01$, ***$p < 0.001$.

may not be involved in HBV replication. Intravenously administered unmodified gapmers are easily excreted by the kidneys owing to the low plasma protein binding efficacy.[32,33] Unmodified gapmers have a very short elimination half-life of several hours.[34] Among the modified gapmers used in this study, phosphorothioated gapmers may require more than a month to be completely excreted from the body,[34] which is attributable to their high plasma protein binding rate leading to difficulty in passing through the glomerulus. Highly water-soluble gapmers are slowly transported from target tissues to the blood.[33] Phosphorothioate-modified antisense drugs have low membrane permeability and remain in intracellular endosomes; moreover, they are not easily metabolized.[35] These gapmer characteristics were induced by the scramble sequence used as a control, so multiple selections must be made as negative controls. Suboptimal antisense drugs tend to remain in target tissues for long periods, and side effects with repeated administration are thought to be due to nucleic acid-induced immune responses.[36]

HBV is specifically taken up by the hepatocytes followed by the replication. Therefore, the challenge for the development of HBV-targeting nucleic acid medicines is to efficiently reach hepatocytes using a suitable drug delivery system (DDS).[37] We selected LNPs as a DDS to effectively deliver siRNA to the liver.[38] We utilized LNPs used in FDA-approved patisiran, previously designed to deliver siRNA to hepatocytes, which has a pharmacokinetic half-life of 3 days.[39] The sequence of siRNA for transthyretin used in patisiran is modified using 2′OMe.[40] Comparably, we introduced 2′OMe and phosphorothioate modifications to siRNA DOCK11-I and the modified siRNA DOCK11-I suppresses the expression of the *DOCK11* gene more efficiently than the normal one. A combination of LNPs and chemically modified siRNAs showed sustained effectiveness in suppressing *DOCK11* expression *in vivo*. LNP chemically modified siRNA targeting DOCK11 was efficiently taken up into HBsAg- and HBcAg-positive hepatocytes in the liver tissues of chronic HBV infection model mice, indicating the possibility of suppressing cccDNA (Figures S7A and S7B). The challenges in nucleic acid medicine discovery include targeting the liver tissue and the stability of nucleic acids, which can be solved using our nanoparticle-nucleic acid formulation, which ensures sustained drug efficacy. Cellular uptake and stability of nucleic acid molecules change depending on the length of the nucleic acid and its modifications,[41] so the DOCK11 nucleic acid medicine has the potential for future development.

In conclusion, we developed LNP-siRNA DOCK11-I, a nucleic acid-based medicine targeting DOCK11 expression, which reduced HBV cccDNA levels in mouse models of HBV infection. Inhibitors of DOCK11 expression may facilitate suppressing HBV cccDNA during the chronic phase of HBV infection. However, before clinical trials, the mechanisms underlying the DOCK11-mediated increase in HBV cccDNA synthesis need to be elucidated for optimizing the therapeutic impact and avoiding the unanticipated side effects. Combination therapy using reverse transcriptase and DOCK11 inhibitors may be a good treatment option for HBV, which needs to be further validated.[42]

MATERIALS AND METHODS

Cell culture

Huh7 and HepG2.2.15 cells were obtained from the American Type Culture Collection (Manassas, VA) and the Japanese Collection of Research Bioresources Cell Bank (Osaka, Japan). The Huh7 and HepG2.2.15 cell lines were maintained using Dulbecco modified Eagle's medium (Thermo Fisher Scientific, Waltham, MA) supplemented with 10% fetal bovine serum, 100 U/mL penicillin, and 100 μg/mL streptomycin. HUVECs were purchased from Lonza (Walkersville, MD) and maintained using an Endothelial Cell Growth Medium-2 Bullet Kit (Lonza, CC-3162).

Mouse primary hepatocytes

Mouse primary hepatocytes were isolated from 8-week-old male C57BL/6J mice as described previously,[43] using collagenase liver digestion. For this purpose, primary hepatocytes were extracted from a liver cell suspension through the density gradient method using Percoll (Sigma-Aldrich, St. Louis, MO). All experiments were performed at least twice. Freshly isolated primary hepatocytes were suspended in the culture medium and seeded in collagen-coated 6-well plates (Iwaki, Tokyo, Japan) and incubated for 24 h at 37°C in a humidified atmosphere maintaining 5% CO_2.

DNA extraction and real-time PCR

Viral DNA was extracted from human cell lines and mouse liver tissues using a NucleoSpin Tissue Kit (TAKARA Bio, Shiga, Japan). cccDNA was obtained by treating the extracted DNA with T5 exonuclease (New England Biolabs, Boston, MA) at 37°C for 30 min followed by incubation for 5 min at 95°C. Expression of cccDNA and HBV DNA were further investigated using a real-time PCR system (QuantStudio 12 K Flex; Thermo Fisher Scientific), specific TaqMan TAMRA or MGB probes, and primers. HBV DNA was detected using 5′-FAM-TATCGCTGGATGTGTCTGCGGCGT-TAMRA-3′, forward: 5′-ACTCACCAACCTCCTGTCCT-3′ and reverse: 5′-GACAAACGGGCAACATACCT-3′. The cccDNA was detected using 5′-FAM-CTGTAGGCATAAATTGGT-MGB-3′, forward: 5′-CGTCTGTGCCTTCTCATCTGC-3′ and reverse: 5′-GCA CAGCTTGGAGGCTTGAA-3′.

RNA extraction, reverse transcription, and real-time PCR

To evaluate the expression of *DOCK11* in mice and humans, total RNA was extracted from human and mouse cells, and mouse liver tissues. Total RNA was extracted from the cell lines using the ISOSPIN Cell and Tissue RNA Kit (Nippon Gene, Tokyo, Japan), whereas the RNeasy Mini Kit (QIAGEN, Valencia, CA) was used to extract total RNA from mouse liver tissue. Complementary DNA (cDNA) was synthesized using the High-Capacity cDNA Reverse Transcription Kit (Thermo Fisher Scientific) following the protocol provided by the manufacturer. Gene expression analysis was conducted using TaqMan probes for human *DOCK11* (Hs00376176_m1), human *DOCK9* (Hs00324508_m1), human *GAPDH* (Hs99999905_m1), mouse *Dock11* (Mm01297557_m1),

and mouse *Gapdh* (Mm99999915_g1) on a QuantStudio 12K Flex real-time PCR system (Thermo Fisher Scientific).

Antisense LNA gapmer

Target sequences matching the antisense LNA gapmer designed for the human DOCK11 mRNA sequences between 3,369 and 3,819 bp were searched using Exicon's algorithm (http://www.exiqon.com/gapmer; Exicon, Skelstedet, Denmark). Three antisense LNA gapmers targeting different regions of the human DOCK11 mRNA (#1: 3,778–3,792 bp; #2: 3,580–3,595 bp; and #3: 3,618–3,633 bp) were selected and synthesized using Exiqon for further *in vivo* and *in vitro* analyses. The sequences of gapmers are as follows: Scramble: 5′-AACACGTC TATACGC-3′, DOCK11#1: 5′-TGTAGAACTCTGTCG-3′, DOC K11#2: 5′-TGGATGCAGAATTAGG-3′, DOCK11#3: 5′- ATTGGC AGGTGAAGTA-3′.

DNA/RNA heteroduplex oligonucleotides (hetero-gapmer)

DOCK11 antisense LNA gapmer and its cRNA with α-tocopherol conjugation at its 5′ end were synthesized by Gene Design (Osaka, Japan). Before *in vitro* and *in vivo* experiments, DOCK11 antisense LNA gapmer and α-tocopherol-conjugated cRNA were mixed in a 1:1 ratio and incubated at 95°C for 5 min followed by slow cooling of the generated DNA/RNA heteroduplex oligonucleotides to reach room temperature. Gapmer negative control 15 bp: 5′-A(L)^A(L)^c^a^c^g^t^c^t^a^t^A(L)^5(L)^G(L)^5(L)-3′, gapmer DOCK11#1 15 bp: 5′-T(L)^G(L)^t^a^g^a^a^c^t^c^t^G(L) ^T(L)^5(L)^G(L)-3′, gapmer negative control 13 bp: 5′-A(L)^A(L) ^c^a^c^g^t^c^t^a^t^A(L)^5(L)-3′, gapmer DOCK11#1 13 bp: 5′-T(L)^G(L)^t^a^g^a^a^c^t^c^t^G(L)^T(L)-3′, gapmer DOCK 11#1 17 bp: 5′-T(L)^T(L)^g^t^a^g^a^a^c^t^c^t^g^T(L)^5(L) ^G(L)^G(L)-3′, negative control 15 bp cRNA + tocophrol: 5′-G(M) ^C(M)^G(M)^U(M)^AUAGACGU^G(M)^U(M)^U(M)-3′, DOC K11#1 15 bp cRNA + tocophrol: 5′-C(M)^G(M)^A(M)^C(M) ^AGAGUUCU^A(M)^C(M)^A(M)-3′ were used for further analyses.

siRNA synthesis and LNP-siRNA preparation

Stealth RNAi siRNA Negative Control Low GC (12935200, Thermo Fisher Scientific) was used as control siRNA. The sense and antisense strands of DOCK11 siRNA were synthesized by Japan Bio Services (Saitama, Japan). The sequences of siRNAs used in this study are as follows: siRNA DOCK11-I S: 5′-UACCCGACAGAGUUCUACA [dT][dT]-3′, AS: 5′-UGUAGAACUCUGUCGGGUA[dT][dT]-3′. siRNA DOCK11-II S: 5′- GGGCUAAAGUCAAAUUUCA[dT] [dT]-3′, AS: 5′- UGAAAUUUGACUUUAGCCC[dT][dT]-3′. Furthermore, nucleic acid modifications such as 2′-O-metylation and phosphorothioate were introduced by Japan Bio Services. Double-stranded siRNA was obtained by mixing equal amounts of the sense and antisense strands, followed by heating at 95°C for 5 min, and gradual cooling to room temperature to facilitate duplex formation. LNPs and DOCK11-targeting LNP-siRNAs were supplied by Nitto (Osaka, Japan). The composition of the LNP was the same as that of patisiran.[21]

Immunohistochemical assay

Formalin-fixed, paraffin-embedded liver tissues were investigated using immunohistochemical (IHC) staining. For this purpose, tissues were deparaffinized, rehydrated followed by antigen retrieval and protein blocking (Protein Block Serum Free; Dako, Carpinteria, CA); subsequently, primary antibodies were applied to the sample slides and incubated overnight at 4°C. DAB color development was performed following the protocol provided with the EnVision+ Kit (Dako). IHC staining images were acquired using a BIOREVO BZ-X810 fluorescence microscope (Keyence, Osaka, Japan). Mouse monoclonal antibodies HBsAg A10F1 (BioLegend, Tokyo, Japan) and HBcAg C1 (Abcam, Cambridge, UK) were used to detect HBsAg and HBcAg, respectively.

AAV8-HBV1.3mer-infected C57BL/6J mice

All animal experiments were approved by the Ethics Committee for the Care and Use of Laboratory Animals, Kanazawa University, Takara-machi Campus, and conducted following the ARRIVE guidelines 2.0. All experiments were performed according to the relevant guidelines and regulations. AAV8-HBV1.3mer virus solution (1×10^{13} vg/mL) was supplied by SignaGen Laboratories (Rockville, MD), which was diluted with physiological saline solution and used to inject 10-week-old male C57BL/6J mice (Jackson Laboratory Japan, Kanagawa, Japan) (1×10^{10} vg/100 μL/mouse) into the tail vein. The mice were housed under specific pathogen-free conditions with a 12-h light/dark cycle and provided *ad libitum* access to tap water and food.

HBV-infected humanized mice

cDNA-uPA wild/+/SCID mice were transplanted with human hepatocytes by Phoenix Bio (Hiroshima, Japan) and infected with diluted hepatitis B virus PBB004 (genotype C). A viral suspension was injected (1×10^5 copies/100 μL/mouse) into the tail vein of mice; the mice were used as a chronic HBV infection model 8 weeks after infection. Humanized mice with human hepatocytes used in all experiments were generated from the same donor (catalog no. 454551, lot no. 195, donor no. HF284). Serum HBsAg and HBeAg levels were measured using the CLEIA method (SRL, Tokyo, Japan). Blood h-Alb levels were measured through a Latex Agglutination turbidimetric immunoassay using an automatic analyzer (Bio Majesty TM; JAC-BM6050, JEOL, Tokyo, Japan).

Method of measuring ALT and AST levels

Serum ALT and AST levels were determined using the Transaminase CII-Test Wako kit (Fujifilm, Osaka, Japan) following the manufacturer's instructions.

Cleavage efficiency assay for siRNA against DOCK11 mRNA

Concatemerization of siRNA target sequences was performed following the protocol described in the 5′-Full RACE Core Set (TAKARA). Specific Human DOCK11 cDNA was synthesized from mRNA by reverse transcription using the 5′ end phosphorylated RT-primer 5′-ATCATCCCAGCATC-3'. RNA was degraded from the hybrid DNA-RNA by treatment with RNase H. Single-stranded

cDNA was concatemerized using T4 RNA Ligase. PCR was performed twice using PrimeSTAR MAX DNA polymerase (TAKARA) using the concatemerized cDNA as a template. The first PCR primer sequence was forward: 5′- TTGCAAGCATCACT TCTTGG -3′ and reverse: 5′- GTGCCTGGTGGAAAATGTCT -3′, and the second sequence was forward: 5′- CCGCTTATGGGTCTT TTCAA -3′ and reverse: 5′- TGCGATTTTCGGTCTATTCC -3'.

Hirt protein-free DNA extraction and Southern blotting to measure the amounts of HBV rcDNA and cccDNA

Linearized pSPT19-full-length HBV was utilized to generate digoxigenin UTP-labeled single-stranded RNA probes following the protocol described in the DIG RNA labeling kit (Roche, Basel, Switzerland).[44] Hirt protein-free DNA was electrophoresed on a 1.2% agarose gel and transferred onto Hybond-N+ membrane (GE Healthcare, Amersham, UK). Hybridization was performed overnight at 50°C in 10 mL DIG Easy Hyb buffer (Roche) containing DIG-labeled single-stranded RNA probes. It was treated with 4 μL of anti-digoxigenin-AP conjugate (Roche) and detected using 1 mL of CSPD (Roche). Image acquisition was performed on a ChemiDoc Touch Imaging System (Bio-Rad, Hercules, CA).

Immunoblotting

Protein extracts were prepared by dissolving mouse liver tissues in RIPA Lysis Buffer (Merck Millipore, Burlington, MA) containing Protease Inhibitor Cocktail and Phosphatase Inhibitor Tablets (Roche Applied Science, Pleasanton, CA). DOCK11 expression was evaluated using rabbit anti-DOCK11 antibody (Bethyl Laboratories, Waltham, MA).

Statistical analysis

Experimental data were analyzed using GraphPad Prism 7 (GraphPad Software, San Diego, CA), and p values were examined using a two-sided unpaired t test with Welch's correction and one-way ANOVA for the *in vitro* experimental results. For the *in vivo* experimental results, p values were determined using the Mann-Whitney U test and a two-sided unpaired t test with Welch's correction. The statistical significance was considered at $p < 0.05$.

DATA AND CODE AVAILABILITY

The data supporting the findings of this study are available from the corresponding authors upon request.

SUPPLEMENTAL INFORMATION

Supplemental information can be found online at https://doi.org/10.1016/j.omtm.2024.101289.

ACKNOWLEDGMENTS

We thank Nami Nishiyama, Mai Nakayama, and Yui Kawamura for their technical assistance in this study. This study was supported by grants approved by the Japan Agency for Medical Research and Development (AMED), grant nos. JP21fk0310110,JP22fk0310514, JSPS KAKENHI, and JP23H00554.

AUTHOR CONTRIBUTIONS

H.O., T. Sakamoto., M.S., S.K., and T.Y. designed the study. H.O., K.N., Y.L., K.K., S.S., T. Shimakami., and N.D. performed the experiments and analyzed the data. H.O., T. Sakamoto., M.H., M.S., S.K., and T.Y. analyzed data. H.O. and T. Sakamoto. wrote the manuscript. All authors contributed to the preparation of the manuscript and provided feedback.

DECLARATION OF INTERESTS

The authors declare no competing interests.

REFERENCES

1. Guvenir, M., and Arikan, A. (2020). Hepatitis B Virus: From Diagnosis to Treatment. Pol. J. Microbiol. *69*, 391–399. https://doi.org/10.33073/pjm-2020-044.
2. Mak, L.Y., Wong, D.K.H., Pollicino, T., Raimondo, G., Hollinger, F.B., and Yuen, M.F. (2020). Occult hepatitis B infection and hepatocellular carcinoma: Epidemiology, virology, hepatocarcinogenesis and clinical significance. J. Hepatol. *73*, 952–964. https://doi.org/10.1016/j.jhep.2020.05.042.
3. Bray, F., Ferlay, J., Soerjomataram, I., Siegel, R.L., Torre, L.A., and Jemal, A. (2018). Global cancer statistics 2018: GLOBOCAN estimates of incidence and mortality worldwide for 36 cancers in 185 countries. CA Cancer J. Clin. *68*, 394–424. https://doi.org/10.3322/caac.21492.
4. Revill, P.A., and Locarnini, S.A. (2016). New perspectives on the hepatitis B virus life cycle in the human liver. J. Clin. Invest. *126*, 833–836. https://doi.org/10.1172/JCI86650.
5. El-Sayed, M.H., and Feld, J.J. (2022). Vaccination at the forefront of the fight against hepatitis B and C. Nat. Rev. Gastroenterol. Hepatol. *19*, 87–88. https://doi.org/10.1038/s41575-021-00570-x.
6. Nassal, M. (2015). HBV cccDNA: viral persistence reservoir and key obstacle for a cure of chronic hepatitis B. Gut *64*, 1972–1984. https://doi.org/10.1136/gutjnl-2015-309809.
7. Marchetti, A.L., and Guo, H. (2020). New Insights on Molecular Mechanism of Hepatitis B Virus Covalently Closed Circular DNA Formation. Cells *9*, 2430. https://doi.org/10.3390/cells9112430.
8. Hashimoto, S., Shirasaki, T., Yamashita, T., Iwabuchi, S., Suzuki, Y., Takamura, Y., Ukita, Y., Deshimaru, S., Okayama, T., Ikeo, K., et al. (2021). DOCK11 and DENND2A play pivotal roles in the maintenance of hepatitis B virus in host cells. PLoS One *16*, e0246313. https://doi.org/10.1371/journal.pone.0246313.
9. Nishikimi, A., Meller, N., Uekawa, N., Isobe, K.i., Schwartz, M.A., and Maruyama, M. (2005). Zizimin2: a novel, DOCK180-related Cdc42 guanine nucleotide exchange factor expressed predominantly in lymphocytes. FEBS Lett. *579*, 1039–1046. https://doi.org/10.1016/j.febslet.2005.01.006.
10. Meller, N., Irani-Tehrani, M., Kiosses, W.B., Del Pozo, M.A., and Schwartz, M.A. (2002). Zizimin1, a novel Cdc42 activator, reveals a new GEF domain for Rho proteins. Nat. Cell Biol. *4*, 639–647. https://doi.org/10.1038/ncb835.
11. Li, Y.Y., Kuroki, K., Shimakami, T., Murai, K., Kawaguchi, K., Shirasaki, T., Nio, K., Sugimoto, S., Nishikawa, T., Okada, H., et al. (2023). Hepatitis B Virus Utilizes a Retrograde Trafficking Route via the Trans-Golgi Network to Avoid Lysosomal Degradation. Cell. Mol. Gastroenterol. Hepatol. *15*, 533–558. https://doi.org/10.1016/j.jcmgh.2022.10.008.
12. Ide, M., Tabata, N., Yonemura, Y., Shirasaki, T., Murai, K., Wang, Y., Ishida, A., Okada, H., Honda, M., Kaneko, S., et al. (2022). Guanine nucleotide exchange factor DOCK11-binding peptide fused with a single chain antibody inhibits hepatitis B virus infection and replication. J. Biol. Chem. *298*, 102097. https://doi.org/10.1016/j.jbc.2022.102097.
13. Doan, P.T.B., Nio, K., Shimakami, T., Kuroki, K., Li, Y.Y., Sugimoto, S., Takayama, H., Okada, H., Kaneko, S., Honda, M., and Yamashita, T. (2023). Super-Resolution Microscopy Analysis of Hepatitis B Viral cccDNA and Host Factors. Viruses *15*, 1178. https://doi.org/10.3390/v15051178.
14. Thompson, A.P., Bitsina, C., Gray, J.L., von Delft, F., and Brennan, P.E. (2021). RHO to the DOCK for GDP disembarking: Structural insights into the DOCK GTPase

nucleotide exchange factors. J. Biol. Chem. *296*, 100521. https://doi.org/10.1016/j.jbc.2021.100521.

15. Yasuhara, H., Yoshida, T., Sasaki, K., Obika, S., and Inoue, T. (2022). Reduction of Off-Target Effects of Gapmer Antisense Oligonucleotides by Oligonucleotide Extension. Mol. Diagn. Ther. *26*, 117–127. https://doi.org/10.1007/s40291-021-00573-z.
16. Sells, M.A., Chen, M.L., and Acs, G. (1987). Production of hepatitis B virus particles in Hep G2 cells transfected with cloned hepatitis B virus DNA. Proc. Natl. Acad. Sci. USA *84*, 1005–1009. https://doi.org/10.1073/pnas.84.4.1005.
17. Nishina, T., Numata, J., Nishina, K., Yoshida-Tanaka, K., Nitta, K., Piao, W., Iwata, R., Ito, S., Kuwahara, H., Wada, T., et al. (2015). Chimeric Antisense Oligonucleotide Conjugated to alpha-Tocopherol. Mol. Ther. Nucleic Acids *4*, e220. https://doi.org/10.1038/mtna.2014.72.
18. Nishina, K., Piao, W., Yoshida-Tanaka, K., Sujino, Y., Nishina, T., Yamamoto, T., Nitta, K., Yoshioka, K., Kuwahara, H., Yasuhara, H., et al. (2015). DNA/RNA heteroduplex oligonucleotide for highly efficient gene silencing. Nat. Commun. *6*, 7969. https://doi.org/10.1038/ncomms8969.
19. Bian, Y., Zhang, Z., Sun, Z., Zhao, J., Zhu, D., Wang, Y., Fu, S., Guo, J., Liu, L., Su, L., et al. (2017). Vaccines targeting preS1 domain overcome immune tolerance in hepatitis B virus carrier mice. Hepatology *66*, 1067–1082. https://doi.org/10.1002/hep.29239.
20. Zlatev, I., Castoreno, A., Brown, C.R., Qin, J., Waldron, S., Schlegel, M.K., Degaonkar, R., Shulga-Morskaya, S., Xu, H., Gupta, S., et al. (2018). Reversal of siRNA-mediated gene silencing in vivo. Nat. Biotechnol. *36*, 509–511. https://doi.org/10.1038/nbt.4136.
21. Thi, T.T.H., Suys, E.J.A., Lee, J.S., Nguyen, D.H., Park, K.D., and Truong, N.P. (2021). Lipid-Based Nanoparticles in the Clinic and Clinical Trials: From Cancer Nanomedicine to COVID-19 Vaccines. Vaccines (Basel) *9*, 359. https://doi.org/10.3390/vaccines9040359.
22. Wu, S.Y., Yang, X., Gharpure, K.M., Hatakeyama, H., Egli, M., McGuire, M.H., Nagaraja, A.S., Miyake, T.M., Rupaimoole, R., Pecot, C.V., et al. (2014). 2'-OMe-phosphorodithioate-modified siRNAs show increased loading into the RISC complex and enhanced anti-tumour activity. Nat. Commun. *5*, 3459. https://doi.org/10.1038/ncomms4459.
23. Biscans, A., Caiazzi, J., Davis, S., McHugh, N., Sousa, J., and Khvorova, A. (2020). The chemical structure and phosphorothioate content of hydrophobically modified siRNAs impact extrahepatic distribution and efficacy. Nucleic Acids Res. *48*, 7665–7680. https://doi.org/10.1093/nar/gkaa595.
24. Uchida, T., Imamura, M., Hayes, C.N., Hiraga, N., Kan, H., Tsuge, M., Abe-Chayama, H., Zhang, Y., Makokha, G.N., Aikata, H., et al. (2017). Persistent Loss of Hepatitis B Virus Markers in Serum without Cellular Immunity by Combination of Peginterferon and Entecavir Therapy in Humanized Mice. Antimicrob. Agents Chemother. *61*, e00725-17. https://doi.org/10.1128/AAC.00725-17.
25. Choi, H.S.J., Tonthat, A., Janssen, H.L.A., and Terrault, N.A. (2022). Aiming for Functional Cure With Established and Novel Therapies for Chronic Hepatitis B. Hepatol. Commun. *6*, 935–949. https://doi.org/10.1002/hep4.1875.
26. Walther, J., Porenta, D., Wilbie, D., Seinen, C., Benne, N., Yang, Q., de Jong, O.G., Lei, Z., and Mastrobattista, E. (2024). Comparative analysis of lipid Nanoparticle-Mediated delivery of CRISPR-Cas9 RNP versus mRNA/sgRNA for gene editing in vitro and in vivo. Eur. J. Pharm. Biopharm. *196*, 114207. https://doi.org/10.1016/j.ejpb.2024.114207.
27. Smekalova, E.M., Martinez, M.G., Combe, E., Kumar, A., Dejene, S., Leboeuf, D., Chen, C.Y., Dorkin, J.R., Shuang, L.S., Kieft, S., et al. (2024). Cytosine base editing inhibits hepatitis B virus replication and reduces HBsAg expression in vitro and in vivo. Mol. Ther. Nucleic Acids *35*, 102112. https://doi.org/10.1016/j.omtn.2023.102112.
28. Boland, A., Côté, J.F., and Barford, D. (2023). Structural biology of DOCK-family guanine nucleotide exchange factors. FEBS Lett. *597*, 794–810. https://doi.org/10.1002/1873-3468.14523.
29. Kang, N., Liu, J., and Zhao, Y. (2019). Dissociation mechanism of GDP from Cdc42 via DOCK9 revealed by molecular dynamics simulations. Proteins *87*, 433–442. https://doi.org/10.1002/prot.25665.
30. Fan, D., Yang, J., Cronin, N., and Barford, D. (2022). Structural basis for CDC42 and RAC activation by the dual specificity GEF DOCK10. Preprint at bioRxiv *5*, 655. https://doi.org/10.1101/2022.06.15.496229.
31. Tavis, J.E., Zoidis, G., Meyers, M.J., and Murelli, R.P. (2019). Chemical Approaches to Inhibiting the Hepatitis B Virus Ribonuclease H. ACS Infect. Dis. *5*, 655–658. https://doi.org/10.1021/acsinfecdis.8b00045.
32. Amantana, A., and Iversen, P.L. (2005). Pharmacokinetics and biodistribution of phosphorodiamidate morpholino antisense oligomers. Curr. Opin. Pharmacol. *5*, 550–555. https://doi.org/10.1016/j.coph.2005.07.001.
33. Akhtar, S. (2009). Oral delivery of siRNA and antisense oligonucleotides. J. Drug Target. *17*, 491–495. https://doi.org/10.1080/10611860903057674.
34. Ferrari, N.S.,R. (2018). Oligonucleotide-Based Drugs and Therapeutics: Preclinical and Clinical Considerations for Development (John Wiley & Sons).
35. Geary, R.S. (2009). Antisense oligonucleotide pharmacokinetics and metabolism. Expet Opin. Drug Metabol. Toxicol. *5*, 381–391. https://doi.org/10.1517/17425250902877680.
36. Kasuya, T., Hori, S.I., Watanabe, A., Nakajima, M., Gahara, Y., Rokushima, M., Yanagimoto, T., and Kugimiya, A. (2016). Ribonuclease H1-dependent hepatotoxicity caused by locked nucleic acid-modified gapmer antisense oligonucleotides. Sci. Rep. *6*, 30377. https://doi.org/10.1038/srep30377.
37. Kulkarni, J.A., Witzigmann, D., Thomson, S.B., Chen, S., Leavitt, B.R., Cullis, P.R., and van der Meel, R. (2021). The current landscape of nucleic acid therapeutics. Nat. Nanotechnol. *16*, 630–643. https://doi.org/10.1038/s41565-021-00898-0.
38. Huang, Y., Zheng, S., Guo, Z., de Mollerat du Jeu, X., Liang, X.J., Yang, Z., Zhang, H.Y., Gao, S., and Liang, Z. (2022). Ionizable liposomal siRNA therapeutics enables potent and persistent treatment of Hepatitis B. Signal Transduct. Targeted Ther. *7*, 38. https://doi.org/10.1038/s41392-021-00859-y.
39. Jing, X., Arya, V., Reynolds, K.S., and Rogers, H. (2023). Clinical Pharmacology of RNA Interference-Based Therapeutics: A Summary Based on Food and Drug Administration-Approved Small Interfering RNAs. Drug Metab. Dispos. *51*, 193–198. https://doi.org/10.1124/dmd.122.001107.
40. Traber, G.M., and Yu, A.M. (2023). RNAi-Based Therapeutics and Novel RNA Bioengineering Technologies. J. Pharmacol. Exp. Therapeut. *384*, 133–154. https://doi.org/10.1124/jpet.122.001234.
41. Vaillant, A. (2022). Oligonucleotide-Based Therapies for Chronic HBV Infection: A Primer on Biochemistry, Mechanisms and Antiviral Effects. Viruses *14*, 2052. https://doi.org/10.3390/v14092052.
42. Watanabe, T., Hayashi, S., and Tanaka, Y. (2022). Drug Discovery Study Aimed at a Functional Cure for HBV. Viruses *14*, 1393. https://doi.org/10.3390/v14071393.
43. Ootsuji, H., Honda, M., Kaneko, S., Usui, S., Okajima, M., Okada, H., Sakai, Y., Takamura, T., Horimoto, K., and Takamura, M. (2010). Altered hepatic gene expression profiles associated with myocardial ischemia. Circ. Cardiovasc. Genet. *3*, 68–77. https://doi.org/10.1161/CIRCGENETICS.108.795484.
44. Cai, D., Nie, H., Yan, R., Guo, J.-T., Block, T.M., and Guo, H. (2013). A Southern Blot Assay for Detection of Hepatitis B Virus Covalently Closed Circular DNA from Cell Cultures. In Antiviral Methods and Protocols (Springer), pp. 151–161. https://doi.org/10.1007/978-1-62703-484-5_13.

OMTM, Volume *32*

Supplemental information

Lipid nanoparticle-encapsulated DOCK11-siRNA efficiently reduces hepatitis B virus cccDNA level in infected mice

Hikari Okada, Takeharu Sakamoto, Kouki Nio, Yingyi Li, Kazuyuki Kuroki, Saiho Sugimoto, Tetsuro Shimakami, Nobuhide Doi, Masao Honda, Motoharu Seiki, Shuichi Kaneko, and Taro Yamashita

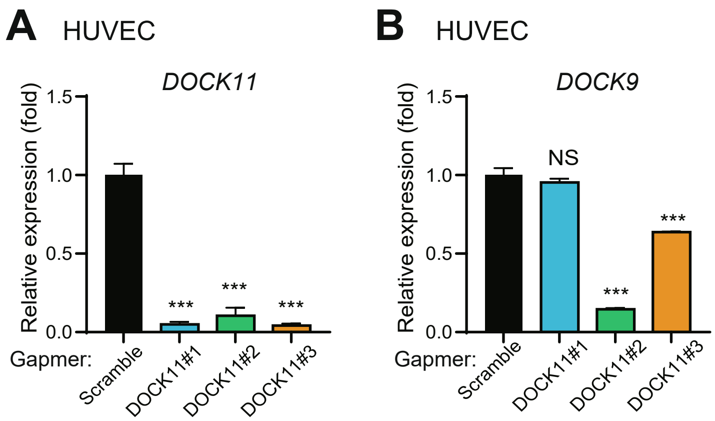

Figure S1. Comparative expression analyses of DOCK11 and DOCK9 in HUVEC after gapmer transfection.
(A, B) Expression levels of DOCK11 (A) and DOCK9 mRNA (B) in HUVEC transfected with gapmer scramble and DOCK11#1-3. Data are presented as the mean (SD) (n = 3) and analyzed using the two-sided unpaired t-test with Welch' s correction. ***P < 0.001.

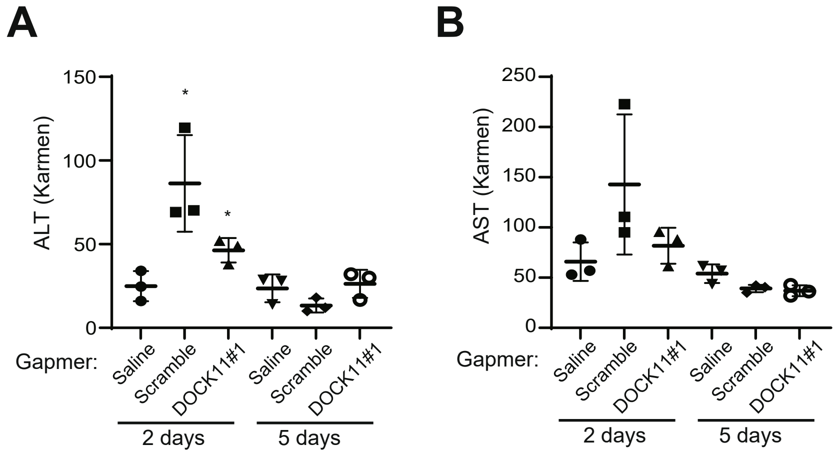

Figure S2. Hepatotoxic effects of gapmers in mice.
(A, B) Serum AST (A) and ALT (B) levels in wild-type mice 2 and 5 days after gapmer injection. Data are presented as the mean (SD) (n = 3) and analyzed using the Mann–Whitney U-test. *P < 0.05.

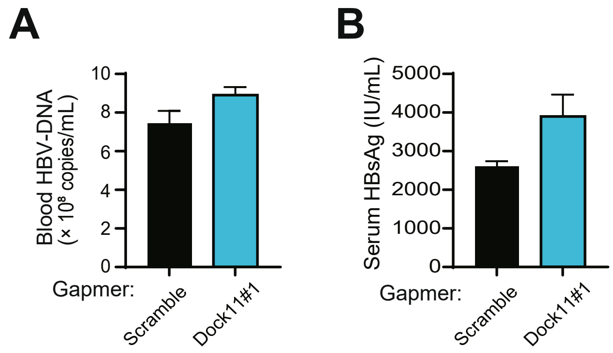

Figure S3. Administration of gapmer DOCK11#1 does not reduce blood HBV-DNA and serum HBsAg levels in HBV-infected human liver chimeric mice. (A, B) Comparative levels of blood HBV-DNA (A) and serum HBsAg (B) in HBV-chronic infected human liver chimeric mice after repeated administration of gapmer scramble and DOCK11#1. Data are presented as the mean (SD) (n = 3).

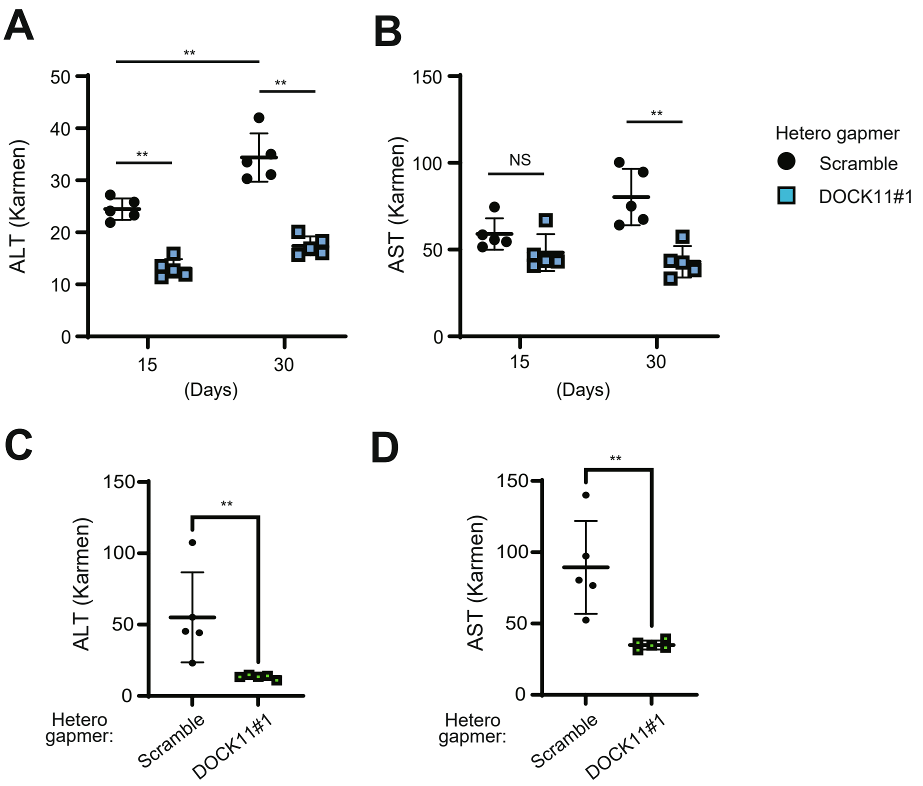

Figure S4. Hepatotoxic effects of hetero-gapmers in wild-type and AAV8-HBV1.3mer-infected mice.
(A, B) Serum ALT (A) and AST (B) levels on day 5 after administration of hetero-gapmer to AAV8-HBV1.3mer infected early and chronic phase model mice.
(C, D) Serum ALT (C) and AST (D) levels in wild-type mice 5 days after hetero-gapmer administration.
Data are presented as the mean (SD) (n = 5) and analyzed using the Mann-Whitney U-test. *P < 0.05, *P < 0.01.

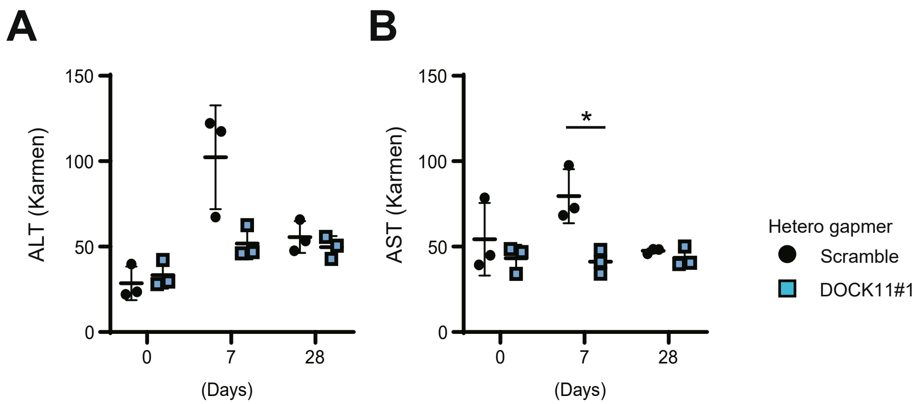

Figure S5. Serum ALT and AST levels to evaluate the hepatotoxicity of hetero-gapmer DOCK11#1 in HBV-infected human liver chimeric mice. (A, B) Comparison of serum ALT (A) and serum AST (B) levels on days 0, 7, and 28 in HBV chronically infected human liver chimeric mice after repeated administration of hetero-gapmer scamble and DOCK11#1. Data are presented as the mean (SD) (n = 3) and analyzed using the Mann-Whitney U-test. *P < 0.05.

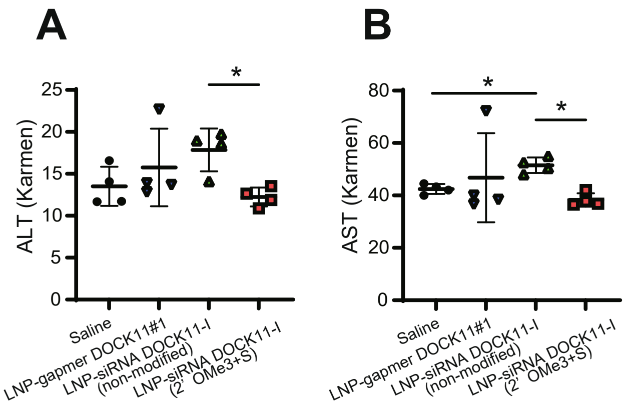

Figure S6. Verification of tropism of LNP-nucleic acid modality in AAV8-HBV1.3mer-infected model mice.
(A, B) Serum ALT (A) and AST (B) levels 7 days after administration of LNP-gapmer, LNP-siRNA non-modified, and LNP-siRNA 2' OMe+S targeting the DOCK11 mRNA in AAV8-HBV1.3mer-infected model mice.
Data are presented as the mean (SD) (n = 4) and analyzed using the Mann-Whitney U-test. *$P < 0.05$.

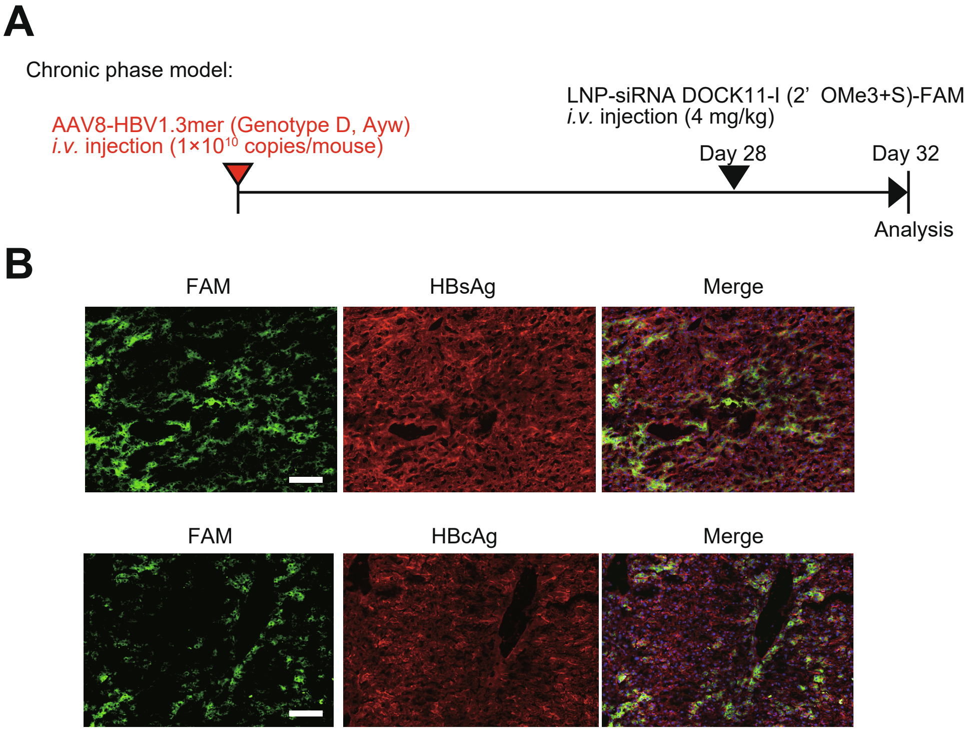

Figure S7. Verification of tropism of LNP-siRNA DOCK11-l in AAV8-HBV1.3mer infected model mice.
(A, B) Schedule for verifying whether LNP-siRNA DOCK11-l (2' OMe3+S) is taken up into HBV-positive hepatocytes of AAV8-HBV1.3mer infection model mice (A). Double fluorescent staining of the liver tissue with Alexa594-labeled HBsAg or HBcAg 5 days after administration of FAM-labeled LNP-siRNA DOCK11-l (B).

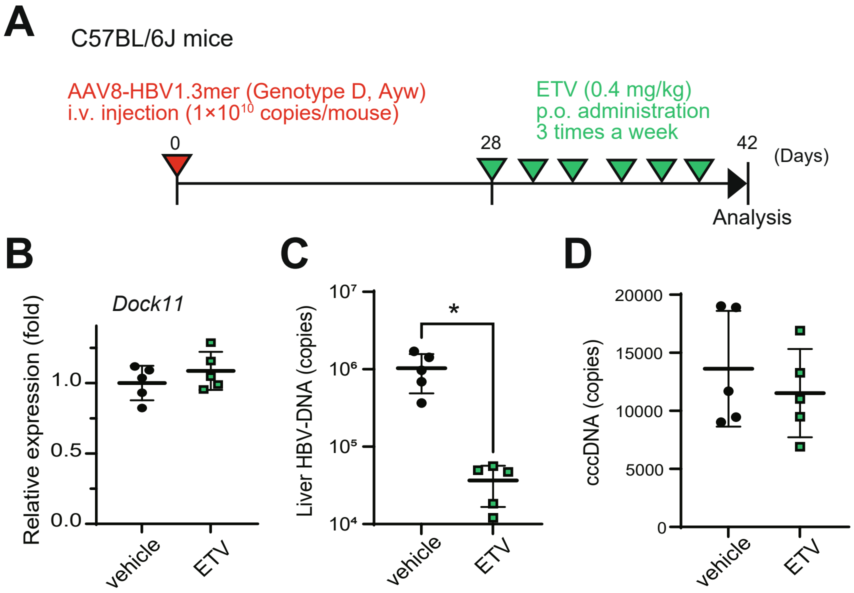

Figure S8. Entecavir (ETV) reduces liver HBV-DNA but not cccDNA levels in AV8-HBV1.3mer infected mice.
(A) The schedule for ETV administration in mouse models of AAV8-HBV1.3mer chronic infection. (B, C, D) The relative expression levels of DOCK11 (B), and HBV DNA (C) and cccDNA (D) levels in the liver of AAV8-HBV1.3mer-infected mice treated with entecavir. Data are presented as the mean (SD) (n = 5) and analyzed using the Mann–Whitney U-test. *$P < 0.05$.

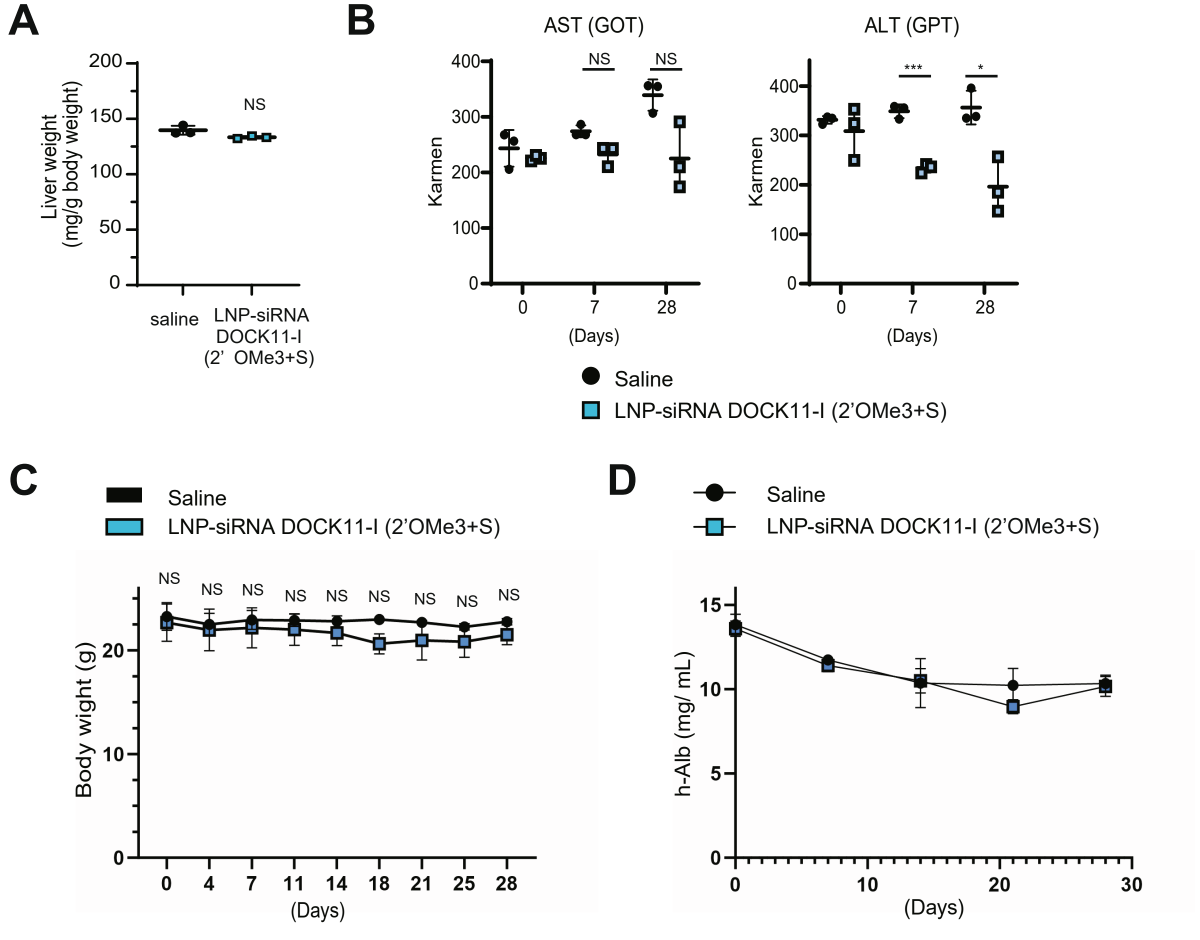

Figure S9. LNP-siRNA DOCK11-I (2’ OMe3+S) exhibits no toxicity in HBV-infected human liver chimeric mice.
The effects of LNP-siRNA DOCK11-I (2’ OMe3+S) on (A, B) the weight of the liver (A) and serum AST and ALT levels (B) in mice.
(C, D) Body weight (C) and serum h-Alb profiles in HBV-infected human liver chimeric mice exposed to repeated treatment with of LNP-siRNA DOCK11-I (2’ OMe3+S).
Data are presented as the mean (SD) (n = 3) and analyzed using the Mann–Whitney U-test. *P < 0.05, ***P < 0.001. NS, not significant.

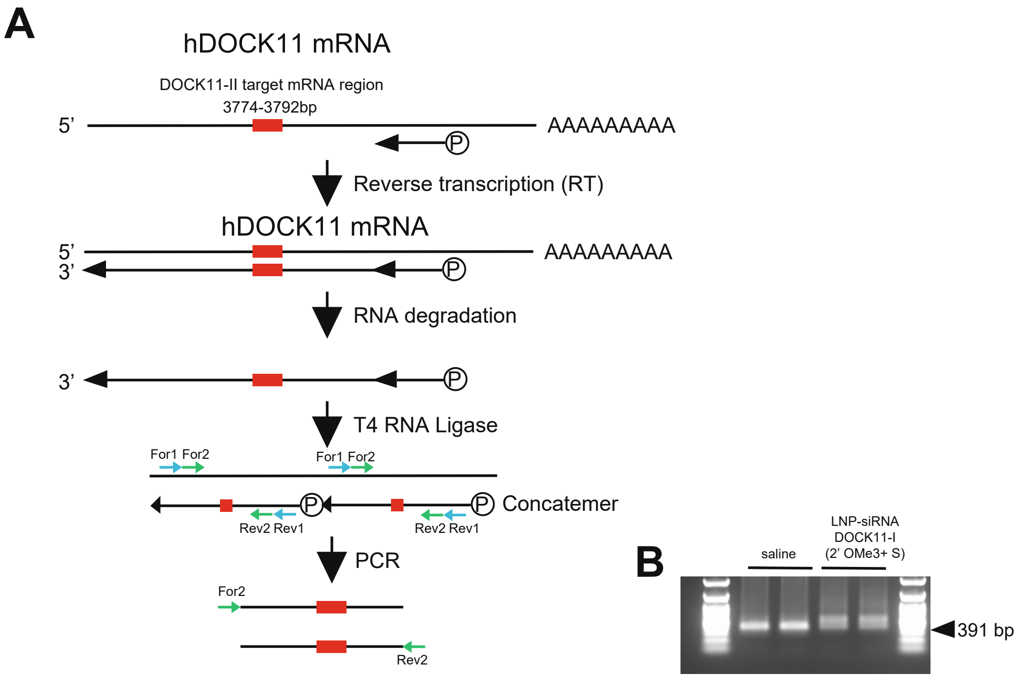

Figure S10. Cleavage efficiency of LNP-siRNA DOCK11-I (2' OMe3+S) against the target sequence of human DOCK11 mRNA in the liver tissue of HBV-infected human liver chimeric mice.

(A) Schematic of evaluating the cleavage efficiency of LNP-siRNA DOCK11-I (2' OMe3+S) against the target sequence of human DOCK11 mRNA.

(B) Cleavage efficiency of human DOCK11 target mRNA in the liver tissues of human liver chimeric mice chronically infected with HBV after repeated administration of Saline and LNP-siRNA DOCK11-I (2' OMe3+S).

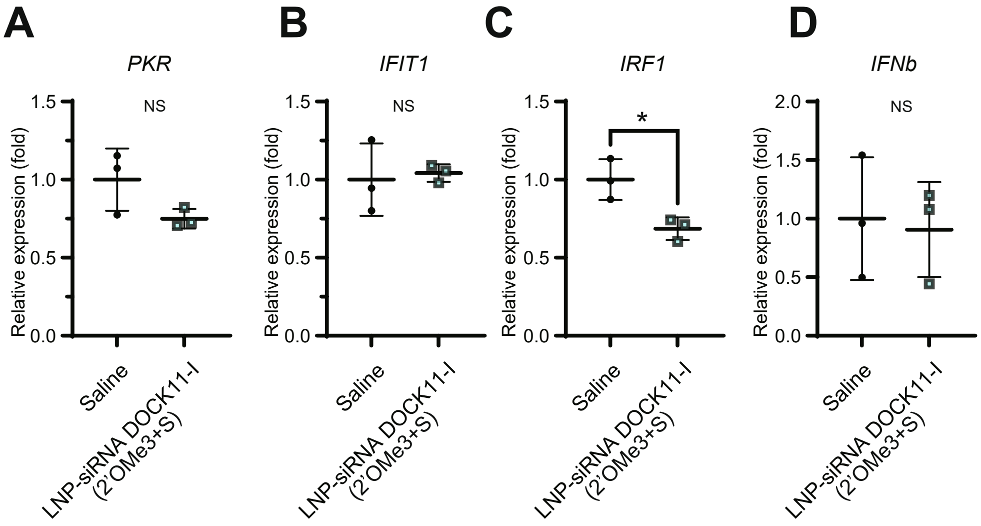

Figure S11. Effect of LNP-siRNA DOCK11-I (2' OMe3+S) on IFN-stimulated genes (ISGs) in HBV-infected human liver chimeric mice.
(A-D) Comparison of gene expression levels of PKR (A), IFIT1 (B), IRF1 (C), and IFNb (D) in human liver chimeric mice chronically infected with HBV after repeated administration of Saline and LNP-siRNA DOCK11-I (2' OMe3+S). Data are presented as mean (SD) (n = 3) and analyzed using the Mann–Whitney U-test. *$P < 0.05$. NS, not significant.
